# Current Practices and a Novel Operational Framework for Planning Research on Digital Health Promotion Interventions From Development to Implementation: Scoping Review

**DOI:** 10.2196/82611

**Published:** 2026-05-06

**Authors:** Claire Collin, Corinne Alberti, Philippe Martin, Graham Moore, Clara Eyraud, Enora Le Roux

**Affiliations:** 1 Université Paris Cité, Inserm, ECEVE Paris France; 2 AP-HP, Hôpital Universitaire Robert Debré Clinical Epidemiology Unit, CIC 1426 Paris France; 3 UR14 - Sexual and Reproductive Health and Rights Institut National d'Etudes Démographiques (INED) Aubervilliers France; 4 Centre for Development, Evaluation, Complexity and Implementation in Public Health Improvement (DECIPHer) School of Social Sciences Cardiff University Cardiff United Kingdom; 5 Wolfson Centre for Young People’s Mental Health Cardiff University Cardiff United Kingdom; 6 University Centre for Adolescent and Young Adult Health, Fondation Santé des Etudiants de France Paris France

**Keywords:** complex interventions, digital health, health promotion, methodological framework, research program design

## Abstract

**Background:**

The UK Medical Research Council’s Guidance on Developing and Evaluating Complex Interventions (MRC GDECI) outlines a 4-phase framework for structuring research programs on interventions: development, feasibility, evaluation, and implementation. However, it provides limited practical direction on how researchers should select which phases to conduct or determine when and whether to progress between phases. This gap is particularly challenging in the context of digital health interventions (DHIs), given their fast-paced and rapidly evolving nature.

**Objective:**

This scoping review examined the research phases conducted, how researchers progressed through them, and the intervention characteristics associated with overall program structure and duration in DHI research, to inform the design of future research programs.

**Methods:**

We searched PubMed, Embase, CINAHL, PsycINFO, and ClinicalTrials.gov to identify complex DHIs promoting health among adolescents and young adults, implemented between 2017 and 2026, for which at least 2 phases of the MRC GDECI were reported, including the evaluation phase. For each eligible intervention, all related protocols, preprints, and published articles were retrieved to reconstruct the full research program. For each program, we analyzed the presence of each research phase, its organization (ie, phase arrangements), and the mechanisms guiding progression between phases (ie, progression mechanisms). Phase-specific and overall program durations were recorded.

**Results:**

A total of 31 research programs, covering 31 interventions and reported in 130 articles, were included. Development, feasibility, evaluation, and implementation phases were reported in 26, 23, 31, and 7 research programs, respectively. Three types of phase arrangements were identified: sequential, iterative, and overlapping. Progression mechanisms between phases included automatic progression, conditional progression based on researchers’ appraisal of findings without prespecified criteria, and progression based on predefined quantitative criteria. Six main research program structures were observed, combining phase arrangements and progression mechanisms. Iterative arrangements were most common, observed in 22 research programs, followed by overlapping (n=10) and strictly sequential structures (n=7). Most progressions relied on researchers’ appraisal of findings without prespecified criteria. Justifications for phase iteration, omission, or progression decisions were rarely reported. The median program duration was 5.8 (IQR 3.8-6.6) years (n=13). Based on these findings, a novel 4-step operational framework and visualization tools were developed to guide the design and planning of DHIs, highlighting key considerations for each step, as well as the strengths, limitations, and risks associated with each phase arrangement and progression mechanism.

**Conclusions:**

This scoping review is the first to systematically examine phase arrangements and progression mechanisms in DHI research programs. Beyond descriptive reporting, it provides a conceptualization of research program structures and offers a flexible operational framework to support the concrete implementation of the MRC GDECI. Greater explicitness in decisions about program structure may enhance methodological rigor, reduce research waste, and improve the integrity and reproducibility of interventions.

**Trial Registration:**

PROSPERO CRD42023401979; https://tinyurl.com/mvc265y3

## Introduction

Complex interventions constitute a significant part of health and social care and require robust methodological approaches to produce reliable evidence for decision-making. To address this, the UK Medical Research Council (MRC) developed the Guidance on Developing and Evaluating Complex Interventions (GDECI), a foundational framework providing theoretical and methodological recommendations across 4 research phases: development, feasibility, evaluation, and implementation [1]. While the MRC GDECI acknowledges that all phases may not be necessary, may not follow a strictly sequential order, and may be revisited when uncertainties persist, it offers limited practical recommendations on which phases to conduct or how to decide when and whether to progress between phases.

This absence of practical methodological direction, while allowing flexible application across diverse interventions, creates challenges for researchers in a context where little research has been conducted on the operationalization of the MRC GDECI in relation to these 2 aspects: phase selection and progression mechanisms between phases.

With regard to phase selection, the boundaries defining what each phase entails have been reported to be insufficiently delineated for the development [2], feasibility [3], and implementation [4] phases, respectively, resulting in blurred distinctions and reduced transparency in reporting [5]. Development phases, in particular, have been shown to be underreported or mixed within feasibility studies, limiting insight into how development processes shape subsequent phases and outcomes [2,5]. Similarly, the proliferation of terminology used to describe feasibility studies has hindered clear differentiation between feasibility, development, and evaluation phases [6]. Persistent ambiguity also remains regarding whether modifications made during feasibility work constitute a return to development or represent adaptations that can appropriately occur within the feasibility phase itself [3]. More broadly, insufficient justification for omitting, merging, or underreporting phases has been highlighted to deprive the scientific community of valuable learning opportunities (both in terms of good practice and failed attempts), compromising transparency, evidence synthesis, and field advancement [7,8], and potentially contributing to research waste [5].

With regard to progression mechanisms between phases, the literature is more developed in some areas, particularly in relation to progression from feasibility to evaluation. Indeed, studies have focused predominantly on the definition and use of predefined quantitative progression criteria to inform decisions about whether to proceed to a full-scale randomized controlled trial (RCT) [9-11]. In contrast, progression decisions in other instances, such as from development to feasibility, from evaluation to implementation, or within feasibility studies that do not rely on quantitative criteria, have received comparatively little attention. The absence of clearly articulated progression mechanisms has been associated with an increased risk of interventions with weaknesses prematurely advancing to later phases, ultimately failing to demonstrate meaningful effects [12-14]. Conversely, prolonged feasibility testing before a full evaluation may impede the timely delivery of evidence to end users, in a context where public health research is expected to produce findings rapidly and efficiently [15,16]. Taken together, the limited documentation of progression mechanisms, particularly beyond feasibility to evaluation, underscores the need for a comprehensive examination of how progression decisions are currently made and reported across intervention research programs.

Among complex interventions, digital health interventions (DHIs) represent a rapidly growing category, typically comprising multiple components addressing simultaneous aims (eg, education, behavior change, or monitoring, and peer engagement) [17,18]. DHIs are increasingly deployed among adolescents and young adults (AYAs), who frequently engage with digital platforms for health information [19]. In this context, DHIs have emerged as promising, innovative, and relatively low-cost approaches to promote healthy behaviors [20,21].

However, the inherently perishable nature of DHIs introduces unique challenges when applying the MRC GDECI. Completing all research phases raises viability concerns, as interventions may become outdated by the time the full research cycle is completed [22]. This is particularly concerning given that traditional phased approaches from intervention development to implementation often span several years, with few interventions ultimately adopted in routine practice [23]. For DHIs targeting AYAs, whose digital preferences [24] and behaviors [25] evolve rapidly, this temporal mismatch between research timelines and technological relevance poses substantial difficulties.

Given the absence of practical guidance on operationalizing the MRC GDECI for different types of complex interventions, notably DHIs, investigation of current research practices is needed to inform complementary guidance. This study, therefore, aimed to examine how researchers conduct phases and progress between them in digital health research. Taking DHIs promoting health among AYAs as a study framework, our specific objectives were to (1) identify the phases conducted in DHI research programs, (2) identify the mechanisms used to progress from one phase to another, (3) quantify the time required to complete programs from development to implementation, and (4) explore the relationship between intervention characteristics and program structure and duration. The ultimate goal of the study is to provide practical guidance on designing and conducting research programs that build on MRC recommendations by being tailored to DHIs.

## Methods

### Study Design

This study is part of a broader project based on a scoping review examining the methodology used to develop and evaluate DHIs for AYAs. The review followed PRISMA-ScR (Preferred Reporting Items for Systematic Reviews and Meta-Analyses extension for Scoping Reviews) guidelines [26] (see Multimedia Appendix 1 for the completed report and abstract checklists). Full methods are described in a prospectively registered PROSPERO (International Prospective Register of Systematic Reviews) protocol (CRD42023401979) and the primary article, which explored the selection, analysis, and interpretation of multiple outcomes in determining intervention success [27]. No deviations from the registered protocol occurred. However, given the breadth and richness of the extracted data, the analyses were structured into 2 complementary articles, each addressing distinct objectives. This study investigates research program design, focusing on phase arrangements, progression mechanisms, and program duration.

### Eligibility Criteria

Eligible interventions were complex DHIs, defined as interventions comprising at least 2 distinct components (eg, videos, quizzes, and informational content) and engaging users throughout at least 2 different modalities, ranging from passive information delivery to interactive engagement with peers and professionals [1,18]. Interventions were required to be delivered exclusively through digital technologies (eg, computer programs, games, mobile apps, social media platforms, and text or voice messages) and to target AYAs, defined as populations with a mean or median participant age between 10 and 24 years, in accordance with the World Health Organization [28]. Eligible interventions had to address health promotion and primary prevention objectives, be implemented between 2017 and 2026, and be published in English or French. The 2017 start date was chosen to ensure the technological relevance of included DHIs in a rapidly evolving digital landscape. This date also coincides with the publication of several seminal papers in late 2016 that established key conceptual and methodological foundations for DHI development and evaluation, with potential downstream influence on intervention design, conduct, and reporting [17,20,22,29-32].

In addition, interventions required preprints or published articles that covered at least 2 of the 4 MRC GDECI phases, whether or not the authors explicitly referred to a phased approach. Phases could be identified through explicit labeling or inferred from commonly used terminology corresponding to the development, feasibility, evaluation, and implementation phases. At least 2 phases were necessary to allow examination of the progression mechanisms between phases. One of these phases had to be the evaluation phase, as the primary article from this review focused on outcome selection and the accuracy of conclusions in that phase. This eligibility criterion was applied to ensure that all 3 study objectives (ie, phase selection, progression mechanisms, and research program duration) could be addressed.

### Information Sources

A total of 4 databases (PubMed, Embase, PsycINFO, and CINAHL) and 1 study registry (ClinicalTrials.gov) were individually searched on February 9, 2023, with updates on May 2, 2023, and January 2, 2024.

For each identified intervention, all associated protocols, published articles, and preprints were retrieved using three complementary approaches: (1) citation tracking using the “Cited By” tool in the PubMed database, (2) reference list screening of included articles, and (3) targeted searches using the intervention name or acronym as a search term across databases, to ensure comprehensive identification of relevant reports.

To capture any additional relevant records related to the included interventions published since January 2, 2024, additional searches were conducted on April 15, 2025, and January 7, 2026. Articles unavailable via open access, university subscriptions, or interlibrary loan were requested directly from the corresponding authors by email, and all eligible articles were successfully retrieved.

### Search Strategy

The search strategy was iteratively developed by the research team in collaboration with the university library and comprised 5 groups of relevant keywords: “eHealth,” “intervention research,” “evaluation,” “health promotion and prevention,” and “adolescents and young adults.” This strategy was applied across all selected databases and registries. The completed PRISMA-S (Preferred Reporting Items for Systematic reviews and Meta-Analyses literature search extension) checklist [33] and detailed search strategies for each database are provided in Multimedia Appendix 2.

### Data Extraction

#### Overview

Title and abstract screening, full-text screening, and data extraction were performed independently by 2 researchers (CC and CE), with discrepancies resolved by a third researcher (ELX). All screening steps were conducted using Covidence systematic review software (Veritas Health Innovation). Data extraction was conducted using EpiData (version 3.1; EpiData Association).

For each research program, data were systematically extracted across 4 domains (see Multimedia Appendix 3 for the data charting form).

#### Phase Arrangements

Using MRC phase definitions [1] and commonly used synonyms in the DHI literature [34], we documented which phases were conducted (development, feasibility, evaluation, and implementation). In addition, we coded whether authors explicitly referenced the MRC GDECI and, if so, in relation to which phase. We also documented whether authors used the term “phase” in a manner consistent with MRC definitions to describe the studies conducted within their research programs, even when the MRC framework was not explicitly cited. Multimedia Appendix 4 details the labels used in the original articles and describes how these were mapped to the corresponding MRC phases.

For each program, we recorded the chronological order of phases as reported by the authors, as well as the presence of phase iteration and phase overlap. Phases were classified as “iterated” when (1) the authors explicitly reported the use of an iterative process during intervention development and (2) multiple pilot studies, efficacy or effectiveness trials, or implementation studies of the same intervention were conducted within the feasibility, evaluation, or implementation phases, respectively. Phases were classified as “overlapping” when (1) for the development and feasibility phases, both were conducted concurrently within a single “formative research” study and reported as a single research output, or when development and piloting activities were interwoven (eg, an initial development stage followed by a pilot study and subsequent redevelopment) and (2) for the evaluation and implementation phases, the authors explicitly reported the use of a hybrid trial design (either Type I or Type II) which by definition simultaneously evaluates the clinical intervention and the implementation strategy [35], or when implementation-related data were collected during the evaluation phase using the same participant sample.

#### Progression Mechanisms

We documented the decision-making processes reported for advancing from one phase to the next, based on authors’ descriptions of methods in the Methods sections and their interpretation of findings in the Discussion sections of the included articles. The following four types of progression situations were identified: (1) authors do not discuss phase-specific results in relation to announced subsequent phases, or progression occurs despite unfavorable or inconclusive findings; (2) authors explicitly discuss phase results and justified progression to the next phase on the basis of positive findings and completion of the current phase, but without predefined progression criteria, relying primarily on a qualitative appraisal of results; (3) authors report the use of prespecified quantitative thresholds, often operationalized through a traffic-light system, to inform progression decisions; and (4) authors do not describe at all the next planned research phase.

#### Duration

Phase-specific and total research program durations were extracted from published articles when reported. Phase-specific duration was defined as the elapsed time between the start and end of each research phase. When phase durations were not reported in the articles, information from trial registries was used as a proxy for phase duration, including ClinicalTrials.gov (“Study Start” and “Study Completion”), International Standard Randomized Controlled Trial Number (ISRCTN; “Date of first enrollment” and “Study completion”), and Australian New Zealand Clinical Trials Registry (ANZCTR; “Date of first participant enrollment” and “Date of last data collection”). This approach was primarily applied to pilot RCTs corresponding to the feasibility phase and to RCTs or quasi-experimental studies corresponding to the evaluation phase, for which trial registrations are typically requested. Total research program duration was defined as the elapsed time from the start of the first phase to the completion of the final phase. Accordingly, total program duration could be calculated for interventions for which both a start date for the first phase and a completion date for the final phase were available, even when phase-specific durations within the research program were not reported.

#### Intervention Characteristics

Several intervention characteristics were recorded including whether the intervention was newly developed or adapted from an existing one (new vs adapted), content was personalized based on participants’ characteristics or standardized (personalized vs standardized), content evolved during delivery or remained fixed (dynamic vs fixed), multiple digital technologies or a single one were used (multiple vs single technology), development was guided by behavioral theory (theory-driven vs empirically-driven), and a participatory approach was used (participatory vs nonparticipatory). We also documented the evaluation study design (RCT vs quasi-experimental design; as these were the only designs used to evaluate eligible interventions) and funding characteristics (amount in US dollars and source: public, private, or mixed).

Consistent with the specific objectives of this scoping review and PRISMA-ScR guidance, no formal critical appraisal of included sources was conducted.

### Data Analysis

Drawing on extracted data, 1 researcher (CC) developed initial typologies of phase arrangements and progression mechanisms, along with operational definitions for each arrangement and mechanism. These typologies were discussed and collaboratively refined with 2 researchers (ELR and CA) until agreement was reached. To assess the reliability of the typologies, 8 (26%) of the 31 research program structures, defined as the specific combination of phase arrangements and progression mechanisms, were independently coded by 2 researchers (CC and CE). Interrater reliability was evaluated using agreement percentages and Cohen κ. The results indicated almost perfect agreement for phase identification, substantial to almost perfect agreement for phase arrangement identification, and substantial to perfect agreement for the identification of progression mechanisms [36]. Detailed interrater agreement percentages and Cohen κ values for each typology category are reported in Multimedia Appendix 5. Following this double coding, the final typologies and corresponding definitions (presented in the Results section) were refined and subsequently applied by CC to derive the program structures of the remaining interventions. Similar structures were grouped and validated by all coauthors.

Phase-specific and total program durations were calculated as medians with first and third quartiles (IQR), expressed in months and years, respectively.

To examine whether researchers tailored programs to intervention-specific characteristics, we explored relationships between intervention characteristics and both program structure and duration through descriptive analyses using counts and percentages only. Further details are provided in Multimedia Appendix 6.

## Results

### Study Selection

**Figure 1:**
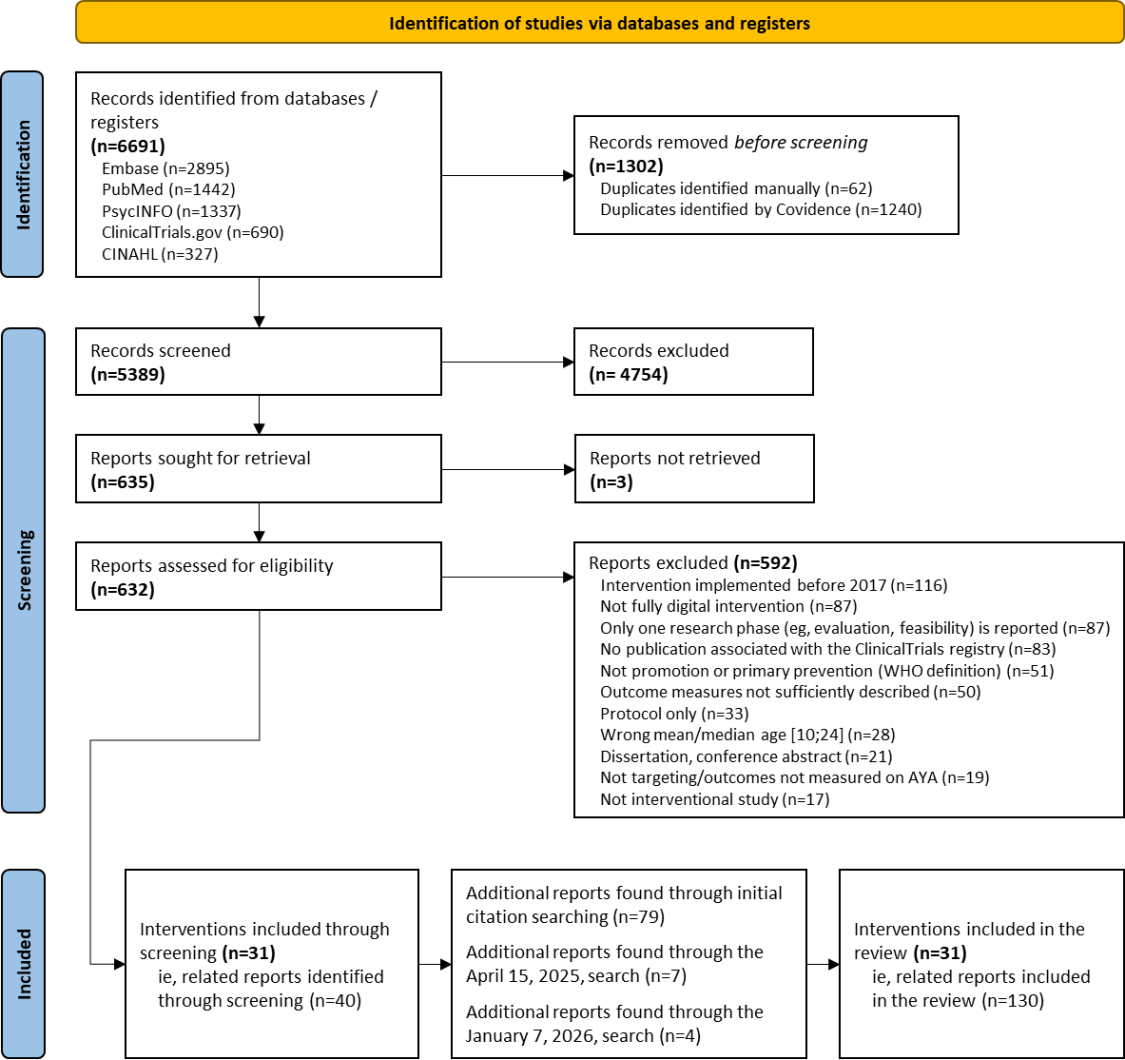
PRISMA (Preferred Reporting Items for Systematic Reviews and Meta-Analyses) flow diagram of the literature search. WHO: World Health Organization. AYA: Adolescent and Young Adult.

The initial searches (February 9, 2023; May 2, 2023; and January 2, 2024) yielded a total of 6691 records, from which 31 interventions, described across 119 articles, met the inclusion criteria. Updated searches restricted to already identified interventions (April 15, 2025, and January 7, 2026) identified 7 and 4 additional articles, respectively. This resulted in a final sample of 31 interventions, with their research programs documented across 130 articles (Figure 1). A median of 4 (IQR 3-6) articles per intervention was included.

### Research Program Characteristics

Of the 31 research programs, 26 included a development phase, 23 a feasibility phase, 31 an evaluation phase (including efficacy or effectiveness evaluation: n=31; process evaluation: n=26; and economic evaluation: n=4), and 7 an implementation phase. One intervention reported a transferability phase, defined as a phase assessing the extent to which positive outcomes observed in a successful intervention, previously evaluated (and implemented) in a primary context, can be replicated in a different target context [37].

In 5 research programs, the MRC GDECI was explicitly referenced, in relation to the development (n=2) and the evaluation (n=3) phases. Four reports highlighted the guidance’s usefulness for intervention development and process evaluation, whereas 1 questioned the applicability of the MRC’s relatively lengthy phased approach to the rapid evolution of digital interventions. A total of 17/31 of the included interventions used the term “phase” in a manner consistent with the MRC GDECI to describe the stages of their research programs.

Research programs were predominantly structured as 3-phase programs, with some following 2- or 4-phase structures (Table 1).

**Table 1:** Description of the research program phases across 31 digital health interventions targeting adolescents and young adults.

Phases		All interventions, n (%)
**2-phase structure**		10 (32)
	Development and evaluation	6 (19)
	Feasibility and evaluation	4 (13)
**3-phase structure**		16 (52)
	Development, feasibility, and evaluation	13 (42)
	Development, evaluation, and implementation	1 (3)
	Feasibility, evaluation, and implementation	2 (7)
**4-phase structure**		5 (16)
	Development, feasibility, evaluation, and implementation	4 (13)
	Development, feasibility, evaluation, and transferability	1 (3)

### Phase Arrangements

The following three types of phase arrangements were observed: sequential, iterative, and overlapping (Table 2).

All interventions used sequential arrangements, with only 7/31 being strictly sequential. The remaining 24/31 combined sequential arrangements with iteration (14/24), overlapping (2/24), or both iteration and overlapping (8/24).

Among the 22 interventions that incorporated iterative arrangements, iteration most commonly occurred during the development (n=17) phase, followed by feasibility (n=5), evaluation (n=1), and implementation (n=1). In 13/22 interventions, the original authors explicitly noted phase iteration as a methodological strength, although the rationale for and frequency of iterations were seldom explained.

**Table 2:** Typology of the phase arrangements used in the digital health intervention research programs.

Phase arrangement	Description	Notation	Visualization
Sequential	Progression is strictly linear from one phase to the next.	>	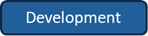
Iterative	One or more phases of the program are repeated at least 2 times.	(i)	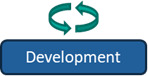
Overlapping	At least 2 phases of the program are conducted simultaneously.	[...+...]	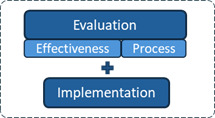

Among the 10 interventions with overlapping arrangements, the most common pattern was “evaluation + implementation” overlap (n=6), followed by “development + feasibility” overlap (n=3), and multiple overlaps (“development + feasibility” and “evaluation + implementation”; n=1).

### Progression Mechanisms

Across the 31 interventions, the following three mechanisms facilitated progression between research phases: automatic progression, conditional progression based on researchers’ appraisal of findings without prespecified criteria, and conditional progression based on predefined quantitative criteria with thresholds (Table 3).

**Table 3:** Typology of progression mechanisms used to move between research phases in digital health research programs.

Progression mechanism^a^	Description	Notation	Visualization
Automatic progression	The next phase begins automatically upon completion of the previous phase, regardless of findings.	*(auto)*	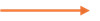
Conditional progression based on researchers’ appraisal of findings without prespecified criteria	Progression is informed by researchers’ appraisal and interpretation of available findings, without reliance on prespecified or clearly reported criteria. Decisions may involve adapting the intervention or methods before progressing.	*(appr)*	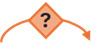
Conditional progression based on predefined quantitative criteria	Progression is informed by preestablished, quantitative criteria (ie, progression criteria). A traffic-light system (or green-amber-red) determines whether to proceed, repeat the phase, or terminate the intervention based on established thresholds.	*(traffic)*	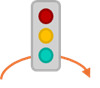

^a^Progression mechanisms that were not reported are represented with the following notation: (NR), and the following visualization: NR. Worked examples: automatic progression: in Wright et al [38], progression from feasibility to evaluation was classified as automatic, as the pilot randomized controlled trial (RCT) of the MIDY intervention showed no intervention effects, yet the research team proceeded directly to a full-scale RCT. Conditional progression based on researchers’ appraisal without prespecified criteria: in Reiter et al [39], progression from feasibility to evaluation for the Outsmart HPV intervention was based on the authors’ narrative appraisal of positive pilot RCT findings (eg, preliminary efficacy and favorable assessment of delivery methods), in the absence of predefined progression criteria or thresholds. Conditional progression based on predefined quantitative criteria: in Garbett et al [40], progression from feasibility to evaluation for the Warna-Warni Waktu intervention was guided by four prespecified quantitative criteria (retention, adherence, data quality, and harm), using a traffic-light system (green=proceed; amber=modify; red=reconsider or terminate), with progression decisions based on observed performance against these thresholds.

A total of 48 phase-to-phase progressions were documented across all included interventions. Among these, the most common mechanism was conditional progression based on researchers’ appraisal of findings without prespecified criteria, accounting for 36/48 mechanisms. Automatic progression was documented in 7 cases: development to feasibility (n=2), development to evaluation (n=1), feasibility to feasibility (n=1), feasibility to evaluation (n=2), and evaluation to implementation (n=1). Only 1 intervention explicitly mentioned the use of predefined progression criteria (traffic-light system) to proceed from feasibility to evaluation. Four mechanisms could not be classified due to insufficient reporting.

### Research Program Structures

By combining phase arrangements and progression mechanisms, the following six groups of program structures were identified and are presented in Table 4: (1) entirely sequential structure, with or without all phases (n=6); (2) entirely sequential structure, with conditional progression based on predefined quantitative criteria (n=1); (3) sequential structure with iterative development (n=9), iterative feasibility (n=3), iterative evaluation (n=1) or iteration of 2 different phases (“development and feasibility”) (n=1); (4) overlapping development and feasibility phases, followed by a sequential structure (n=3); (5) sequential early phases (development and feasibility) followed by overlapping evaluation and implementation (n=6); and (6) structure with multiple overlaps (“development + feasibility” and “evaluation + implementation”; n=1). Multimedia Appendix 7 provides one worked example for each of the 6 research structures, detailing the phase arrangements and progression mechanisms as reported by the authors and classified according to the developed typologies. Multimedia Appendix 8 presents the overall program structure and phase duration for each included intervention.

**Table 4:** Main research program structures identified in 31 digital health interventions.

Structure	Description	Visual description	Notation
Structure 1	Sequential structure only, with automatic progression or progression based on researchers’ appraisal without prespecified criteria (n=6)	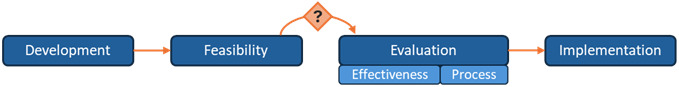	D *(auto)* > F *(appr)* > E *(auto)* > I
Structure 2	Sequential structure only, with progression based on predefined quantitative criteria (n=1)	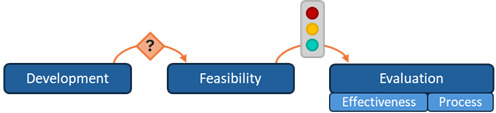	D *(appr)* > F *(traffic)* > E
Structure 3	Iterative development (n=9), iterative feasibility (n=3), iterative evaluation (n=1) or 2-phase iteration (n=1) within a sequential structure, with automatic progression or progression based on researchers’ appraisal without prespecified criteria (n=14)	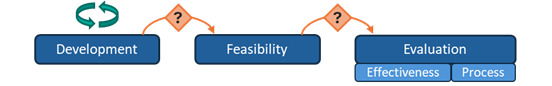	D(i) *(appr)* > F *(appr)* > E
Structure 4	Overlapping development and feasibility phases (with or without iteration) within a sequential structure, with automatic progression or progression based on researchers’ appraisal without prespecified criteria (n=3)	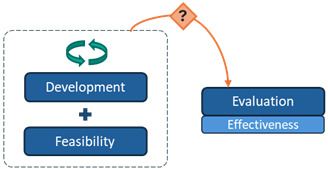	[D(i)+F] *(appr)* > E
Structure 5	Overlapping evaluation and implementation phases (with or without iteration) within a sequential structure, with automatic progression or progression based on researchers’ appraisal without prespecified criteria (n=6)^a^	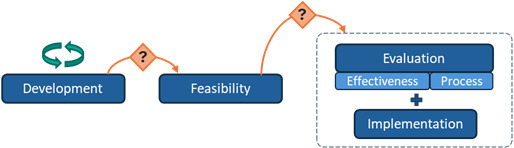	D(i) *(appr)* > F *(auto)* > [E+I]
Structure 6	Multiple overlaps within a sequential structure, with progression based on researchers’ appraisal without prespecified criteria (n=1)	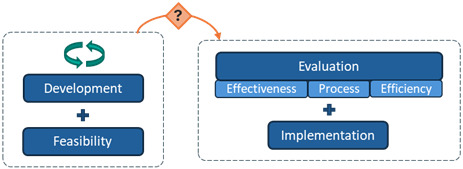	[D(i)+F] *(appr)* > [E+I]

^a^Count (n=6) includes 1 intervention in which a transferability phase replaced the implementation phase. Phases: D: development (n=26); F: feasibility (n=23); E: evaluation (n=31); I: implementation (n=7). Phase arrangements are as follows: >: sequential; (i): iterative; [+]: overlapping. Progression mechanisms: *(auto)*: automatic progression; *(appr)*: conditional progression based on researchers’ appraisal of findings without prespecified criteria; *(traffic)*: conditional progression based on predefined quantitative criteria. Main structures may be further divided into substructures based on the progression mechanisms used to move from one phase to the next. Multimedia Appendix 7 provides a worked example for each main research structure.

### Research Program Duration

The complete research program duration was available for 13 interventions. The median total duration of those programs was 5.8 (IQR 3.8-6.6) years (n=13).

Phase-specific durations were available for varying numbers of interventions. Phase-specific median durations were 18 (IQR 9-31) months for development (n=11); 5 (IQR 1-9) months for feasibility (n=16); 15 (IQR 8-27) months for evaluation (n=30); and 48 (IQR 48-48) months for implementation (n=1).

Cumulative median time for research phases only, excluding gaps between phases, was 3.5 (IQR 2.2-4.4) years (n=13).

### Relationships Between Intervention Characteristics and Program Structure and Duration

Intervention characteristics, along with descriptive statistics and tests of hypotheses between intervention characteristics and program structure and duration, are presented in Multimedia Appendix 9. Exploratory analyses were limited by missing data on funding (15/31, 48%) and duration (13/31, 42%), and small subgroup sizes.

Regarding program structure, newly developed interventions compared to adapted ones (15/19, 79% vs 5/12, 42%) and interventions with dynamic content compared to those with fixed content (4/5, 80% vs 16/26, 62%) showed greater iteration in the early phases. Studies with higher research funding were more complete 3- or 4-phase research programs. Specifically, all 3 interventions with funding over US $5 million used either 3-phase (n=1) or 4-phase (n=2) programs.

Regarding program duration, these well-funded programs tended to have longer evaluation phases (lasting over 24 months).

No patterns were observed between other intervention characteristics and program structure or duration.

### Synthesizing Results: A Novel Operational Framework for Research Program Design

**Figure 2:**
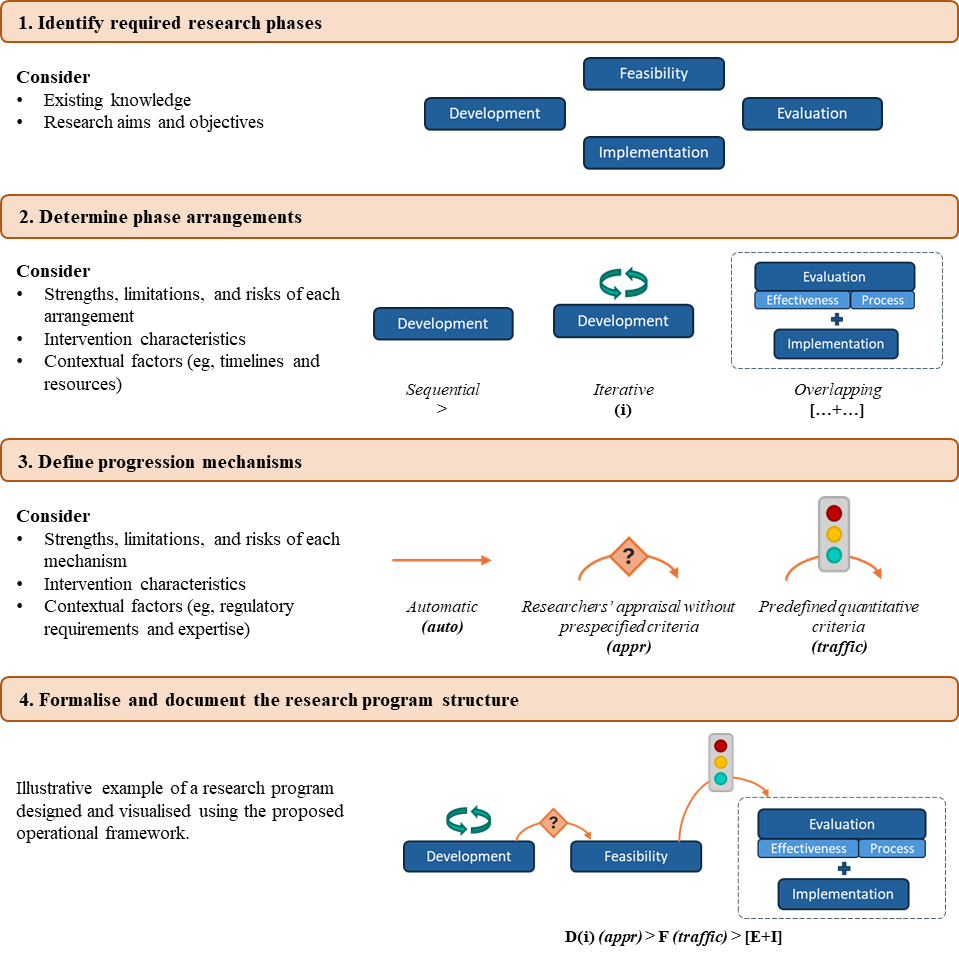
Operational framework for designing and planning digital health promotion interventions in terms of phase arrangements and progression mechanisms.

Building on the findings related to research program characteristics, structure, and duration, and informed by the MRC GDECI, a novel 4-step operational framework was developed to support the planning, reporting, and critical appraisal of DHI research programs (Figure 2).

The framework promotes structured decision-making across 4 sequential steps. Step 1 involves explicit identification of the research phases to be included (ie, development, feasibility, evaluation, and implementation). Step 2 requires specification of how these phases will be arranged (ie, sequentially, iteratively, or in overlapping arrangements). Step 3 concerns the progression mechanisms between phases (ie, automatic progression, progression based on researchers’ appraisal without prespecified criteria, or progression based on predefined quantitative criteria). Finally, Step 4 entails formalization of the overall research program structure using the proposed visual tools and associated notation.

At each step, the framework highlights key considerations for decision-making: research aims guide phase selection, while intervention characteristics (eg, degree of novelty, behavioral complexity, and technological uncertainty) and contextual constraints inform choices regarding phase arrangements and progression mechanisms.

For example, novel interventions incorporating untested technologies or complex behaviors may benefit from iterative development with predefined quantitative criteria to ensure adequate refinement before costly evaluation. Conversely, interventions with established effectiveness may use overlapping phases to accelerate implementation. Regarding progression mechanisms, conditional progression based on researchers’ appraisal of findings without prespecified criteria enables nuanced decision-making but requires transparent documentation of decision criteria and rationale. In contrast, predefined progression criteria maximize transparency and reproducibility but demand careful selection of meaningful metrics and thoughtful interpretation.

To complement the framework, and drawing on both the existing literature and the patterns identified in this review, Table 5 synthesizes the key strengths, potential risks, and methodological limitations of each phase arrangement and progression mechanism [5,13,41-44].

**Table 5:** Strengths, risks, and limitations of the phase arrangements and progression mechanisms identified in digital health interventions research programs.

Strengths				Risks and limitations
**Phase arrangements**				
	Sequential	Provides a clear structure with distinct phase boundaries Ensures systematic progression through research stages (or justification as to why one is omitted) Facilitates reporting, outcome selection, and interpretation, and scientific valorization of each phase Supports targeted funding applications and allocation per phase	May increase total program duration (compared to overlapping arrangements) Limits flexibility to respond to emerging uncertainties (risk of overadherence to preestablished plans) May delay the implementation of promising interventions	
	Iterative	Enables unresolved uncertainties to be addressed before progression Leads to more refined and context-sensitive interventions Supports real-time learning and adaptation throughout the program	Extends the duration of individual phases Increases research costs Requires transparent reporting of rationale and changes at each iteration More complex to design, coordinate, and manage Requires criteria to determine when further iteration is unnecessary or unproductive	
	Overlapping	Enables simultaneous work on multiple phases, potentially reducing total program duration Enables cross-phase interpretation of outcomes while discussing intervention success (notably evaluation and implementation outcomes)	Blurs methodological boundaries between phases Increases complexity in research planning, coordination, and outcome interpretation More resource-intensive (in terms of funding, staffing, expertise, and logistics)	
**Progression mechanisms**				
	Automatic progression	Ensures timely progression between phases, reducing delays Simplifies program planning and management	May allow progression of underdeveloped or unfeasible interventions Risk of propagating early flaws in the intervention into subsequent phases Involves minimal to no decision-making regarding progression	
	Conditional progression based on researchers’ appraisal of findings without prespecified criteria	Enables holistic assessment drawing on multiple data sources Allows integration of both quantitative and qualitative findings Permits context-sensitive decision-making informed by intervention-specific knowledge	Prone to reporting (selective reporting) and confirmation biases Lacks standardization and transparency in decision-making Increases planning complexity Limits reproducibility and comparability across studies	
	Conditional progression based on predefined quantitative criteria (progression criteria)^a^	Ensures transparent and standardized decision-making Provides objective thresholds to determine success Reduces the risk of reporting bias Enhances study transparency, replicability, and reproducibility	Decision-making precedes experimentation, risking overlooking unexpected findings Risk of setting arbitrary or inappropriate thresholds Potential disconnect between metrics and meaningful intervention success Reduces flexibility to adapt to emerging insights	

^a^With respect to the use of predefined quantitative criteria, Mellor et al [13] propose a four-step framework (ie, design, conduct, analysis, and reporting) to guide researchers in the application of such progression mechanisms.

## Discussion

### Summary of Findings

This study examined how DHI research programs align with the MRC GDECI, focusing on how research phases are arranged and how progression between phases occurs. Our analysis of 31 programs revealed no single dominant structure, but several structures characterized by 3 types of phase arrangements (sequential, iterative, and overlapping) and 3 progression mechanisms (automatic, appraisal-based, and predefined criteria). Iteration, particularly of early phases (development and feasibility), and overlaps were common. Most progressions relied on researchers’ appraisal or occurred automatically, with only 1 program using predefined quantitative criteria. Justifications for phase iteration or omission, or the choice of progression mechanisms, were rarely documented, corroborating existing literature [9]. Median program duration was 5.8 (IQR 3.8-6.6) years (n=13), while the median cumulative duration of individual phases was 3.5 (IQR 2.2-4.4) years (n=13). Finally, a novel, 4-step operational framework, along with the strengths, potential risks, and methodological limitations of each phase arrangement and progression mechanism, is proposed to support researchers in making informed decisions in designing their DHI research programs.

### Interpretation and Implications of Findings

#### Phase Arrangements

Strictly sequential arrangements, arguably aligning most closely with a traditional MRC GDECI interpretation, were relatively uncommon. This may reflect differential guidance application in response to intervention-specific requirements.

Iterative arrangements were frequent, likely reflecting the dynamic nature of DHIs [20]. These interventions often require multiple rounds of user testing, feedback incorporation, and technological refinement [17], consistent with agile and user-centered methodologies increasingly promoted in digital research [45-47]. Iterative approaches may be particularly relevant for DHIs targeting AYAs, to sustain engagement among digitally savvy participants with elevated expectations for sophisticated technologies as found in gaming and social media [48,49]. However, repeated early-phase iterations may prolong research timelines and increase costs [50], necessitating a balance between enhancing relevance and mitigating the risk of obsolescence in rapidly evolving digital environments [22].

The use of overlapping phases, while not explicitly endorsed in the MRC GDECI, reflects emerging interests in hybrid research designs intended to accelerate translation from evaluation to implementation [35]. For DHIs, such arrangements may provide simultaneous insights into multiple research phases while addressing rapid technological evolution [41]. However, hybrid research designs require advanced methodological expertise to prevent evaluation and implementation of underdeveloped interventions [51], potentially increasing research waste. The rise of overlapping arrangements also suggests that the strict phase boundaries outlined in the MRC GDECI may not fully reflect the realities of DHI research. This observation supports the need for more flexible, context-sensitive adaptations of the guidance, as has already been advocated in other fields, including palliative care and rehabilitation research [52-54].

#### Progression Mechanisms

The important reliance on conditional progression based on researchers’ appraisal of findings without prespecified criteria and automatic progression may stem from several factors. First, current funding structures often support evaluation-focused programs [55], potentially incentivizing progression regardless of early-phase outcomes. Second, predefined progression criteria, originally formalized within clinical research, have probably not yet been adopted in DHI research; as Perski and Short [56] have, for instance, shown for DHI acceptability assessment, for which thresholds are rarely specified, and, when present, are typically not grounded in clear theoretical or empirical justification. Third, the complexity of DHIs may preclude reliance on simple, predefined quantitative thresholds to determine phase completeness [17].

Nevertheless, the limited use of predefined progression criteria is noteworthy given their endorsement in methodological literature for enhancing research integrity and transparency, while limiting biases [9,13]. This gap is especially problematic given the risk of inadequate documentation or underreporting of progression decisions when explicit criteria are not used [11]. However, the use of such criteria warrants critical attention: many lack an objective scientific basis, and their overmechanistic use may prove problematic [10].

#### Program Duration

The median program duration of 5.8 (IQR 3.8-6.6) years is substantially shorter than the often-cited 17-year development to implementation timeline for complex health care innovations [23], potentially reflecting inherently faster DHI development driven by competitive pressures and the relative ease of digital deployment compared to face-to-face interventions [22]. However, several alternative explanations may account for this observed difference. These include the limited number of implementation studies in our sample, the substantial amount of missing duration data, and our reliance on trial registry dates to approximate phase-specific duration. The latter approach may have underestimated phase duration, particularly when the “date of last data collection” was the only available proxy for phase completion. In practice, the activities required to formally conclude a phase, such as data analysis, interpretation of findings, decision-making regarding progression to subsequent phases, and publication, often extend for several additional months and, in some cases, years beyond the end of data collection [57].

While shorter timelines appear advantageous for DHIs, the gap between total program duration and cumulative phase duration (5.8 vs 3.5 years, respectively) still reveals substantial interphase delays, likely due to funding acquisition, ethical approvals, or regulatory processes [58]. Such delays are particularly concerning for DHIs given the risk of rapid technological obsolescence [22].

#### Implications and Applicability of the Operational Framework

The developed framework, alongside the identified strengths, potential risks, and limitations of phase arrangements and progression mechanisms, represents one of the main contributions of this review. It is designed to support researchers in making transparent, reflexive, and methodologically informed decisions when structuring research programs and defining progression between phases. Importantly, it is not intended as a prescriptive or universal model. Rather, it provides a set of “building blocks,” namely, phase arrangements and progression mechanisms, that can be selected, combined, and adapted according to intervention characteristics, contextual realities, and practical constraints, provided that such selections are explicitly justified and transparently reported.

While based on the MRC GDECI and developed from DHI-specific findings, the developed framework has potential for applicability beyond the guidance and DHIs to prevention and health promotion interventions more broadly. While our results suggested that not all authors used the MRC GDECI, phased approaches to structuring research programs were explicitly reported in more than half of the interventions and could be inferred in all included DHIs. Providing operational guidance for more transparent arrangements and progressions between research phases may therefore benefit a wide range of researchers, including those who do not explicitly reference the MRC GDECI.

The framework is also likely applicable to non-DHI interventions, although the resulting research structures may differ, as DHIs and non-DHIs have distinct characteristics. DHIs may require shorter timelines and benefit from iterative early phases to produce engaging, user-ready tools. In contrast, more stable health promotion interventions, such as school-based programs, may support longer, sequential programs with a greater emphasis on implementation. These differences highlight the potential for adapting the framework to diverse intervention types while retaining its core principles for phased research program structuring.

### Strengths and Limitations

This study is the first to systematically examine DHI research program structures against established methodological guidance, offering valuable insights into current practices. The proposed framework turns these findings into a practical, visual tool to assist with future research planning and funding decisions.

Limitations must be acknowledged. Exploratory analyses of the relationships between intervention characteristics and program structure and duration were constrained by missing data on funding and duration, and by small sample sizes within subgroups of intervention characteristics and phase arrangements. Although the absence of clear patterns in our analyses could be interpreted as a lack of relationships between the selected intervention characteristics and program structure or duration, it is equally plausible that limited sample sizes hindered the detection of such relationships. As a result, future research with larger datasets is needed to assess these relationships more robustly and to support the development of additional propositions and recommendations to better guide research program design. Furthermore, the relatively small number of implementation studies identified in the review raises questions regarding the chosen eligibility criteria and search strategies. With respect to eligibility criteria, requiring included interventions to report at least 2 research phases and an evaluation component may have led to the underrepresentation of single-phase studies, nonevaluated pilots, and pilots that were discontinued early due to negative feasibility findings or lack of further funding. Although this criterion was necessary to document progression mechanisms, it privileged research programs structured around an evaluation phase. Nevertheless, this focus remains highly relevant, as the effectiveness of interventions is assessed during the evaluation phase and constitutes a critical stage in intervention development and testing. With respect to search strategies, the focus on academic databases may have led to the overlooking of some implementation studies published outside peer-reviewed journals or described using terminology not captured by the search strategy. This may have limited our ability to provide a more comprehensive account of the conduct, arrangement, and progression toward real-world implementation phases within research programs. However, this pattern may also reflect broader structural challenges in conducting and publishing implementation research, including funding mechanisms that prioritize development and evaluation over implementation and scale-up.

### Conclusion

To our knowledge, this scoping review is the first to systematically examine how DHI research programs are structured with respect to phase arrangements and progression mechanisms, and to explore how intervention characteristics are associated with program structure and duration. By synthesizing current practices and the decision-making processes underpinning program design, this review advances the field by moving beyond descriptive reporting to offer a conceptualization of research program structures that provides practical direction for the concrete operationalization of the MRC GDECI framework.

The resulting operational framework offers a flexible, nonprescriptive tool that supports transparent, context-sensitive decision-making in research planning, making an original contribution to the field. These findings carry practical implications for researchers, funders, journal editors, and the broader scientific community. Greater explicitness in defining phase arrangements and progression mechanisms may enhance methodological rigor, reduce research waste, and improve the integrity and reproducibility of DHIs, as well as of prevention and health promotion interventions more broadly.

Future research should further test, refine, and validate this framework across diverse intervention types and investigate factors associated with decision-making for phase arrangements and progression mechanisms.

## Appendix

Multimedia Appendix 1 Completed PRISMA-ScR 2019 checklist for report and abstract.

Multimedia Appendix 2 PRISMA-S checklist and search strategies by database.

Multimedia Appendix 3 Data charting form.

Multimedia Appendix 4 Terminology used to name research phases.

Multimedia Appendix 5 Interrater agreement on classifying phase conduct, phase arrangement, and progression mechanisms for 8 digital health interventions.

Multimedia Appendix 6 Hypotheses linking intervention characteristics and research program structure and duration.

Multimedia Appendix 7 Worked examples of digital health research program structures (A-F).

Multimedia Appendix 8 Research program structure, duration, and corresponding references for each intervention included in the study.

Multimedia Appendix 9 Description of intervention characteristics and hypothesis testing.

## Abbreviations

AYA: adolescent and young adult
DHI: digital health intervention
GDECI: Guidance on Developing and Evaluating Complex Interventions
ISRCTN: International Standard Randomized Controlled Trial Number
MRC: UK Medical Research Council
PRISMA-S: Preferred Reporting Items for Systematic reviews and Meta-Analyses literature search extension
PRISMA-ScR: Preferred Reporting Items for Systematic Reviews and Meta-Analyses extension for Scoping Reviews
PROSPERO: International Prospective Register of Systematic Reviews
RCT: randomized controlled trial

## Data Availability

The datasets generated or analyzed during this study are available in the Zenodo repository [59].
