# Supplementary material for: Current Practices and a Novel Operational Framework for Planning Research on Digital Health Promotion Interventions From Development to Implementation: Scoping Review
Source: J Med Internet Res. 2026 May 6;28:e82611. doi: 10.2196/82611 (PMC13191305; doi:10.2196/82611)
Supplement: Multimedia Appendix 2 [file jmir_v28i1e82611_app2.docx]

### Multimedia Appendix 2. PRISMA-S Checklist and Search strategies by database

**PRISMA-S Checklist**

| **Section/topic** | **#** | **Checklist item** | **Location(s) Reported** |
| --- | --- | --- | --- |
| **INFORMATION SOURCES AND METHODS** | | | |
| Database name | 1 | Name each individual database searched, stating the platform for each. | Page 9 |
| Multi-database searching | 2 | If databases were searched simultaneously on a single platform, state the name of the platform, listing all of the databases searched. | Not done. Databases searched individually. See *Methods – Information Sources* section. |
| Study registries | 3 | List any study registries searched. | Page 9 |
| Online resources and browsing | 4 | Describe any online or print source purposefully searched or browsed (e.g., tables of contents, print conference proceedings, web sites), and how this was done. | Page 9 |
| Citation searching | 5 | Indicate whether cited references or citing references were examined, and describe any methods used for locating cited/citing references (e.g., browsing reference lists, using a citation index, setting up email alerts for references citing included studies). | Page 9 |
| Contacts | 6 | Indicate whether additional studies or data were sought by contacting authors, experts, manufacturers, or others. | Page 9 |
| Other methods | 7 | Describe any additional information sources or search methods used. | Page 9. Search by intervention name or acronym is described. |
| **SEARCH STRATEGIES** | | | |
| Full search strategies | 8 | Include the search strategies for each database and information source, copied and pasted exactly as run. | Multimedia Appendix 2 |
| Limits and restrictions | 9 | Specify that no limits were used, or describe any limits or restrictions applied to a search (e.g., date or time period, language, study design) and provide justification for their use. | Multimedia Appendix 2 |
| Search filters | 10 | Indicate whether published search filters were used (as originally designed or modified), and if so, cite the filter(s) used. | Multimedia Appendix 2 |
| Prior work | 11 | Indicate when search strategies from other literature reviews were adapted or reused for a substantive part or all of the search, citing the previous review(s). | The search strategy was original. |
| Updates | 12 | Report the methods used to update the search(es) (e.g., rerunning searches, email alerts). | Page 9 |
| Dates of searches | 13 | For each search strategy, provide the date when the last search occurred. | Page 9 |
| **PEER REVIEW** | | | |
| Peer review | 14 | Describe any search peer review process. | Page 9 |
| **MANAGING RECORDS** | | | |
| Total Records | 15 | Document the total number of records identified from each database and other information sources. | Page 13 + Multimedia Appendix 7 |
| Deduplication | 16 | Describe the processes and any software used to deduplicate records from multiple database searches and other information sources. | Page 9 + Multimedia Appendix 7 |

*From:* Rethlefsen ML, Kirtley S, Waffenschmidt S, Ayala AP, Moher D, Page MJ, Koffel JB; PRISMA-S Group. PRISMA-S: an extension to the PRISMA Statement for Reporting Literature Searches in Systematic Reviews. *Syst Rev*. 2021;**10**(1):39. doi: 10.1186/s13643-020-01542-z.

**Search strategies by database**

**PubMed**

Search history

| Date | Number of references retrieved | Number of duplicates | Number of references added to screen |
| --- | --- | --- | --- |
| February 9^th^ 2023 | 1,349 | 0 | 1,349 |
| May 2^nd^ 2023 | 31 | 4 | 27 |
| January 2^nd^ 2024 | 62 | 6 | 56 |
|  | **1,442** | **10** | **1,432** |

Search terms

(("Telemedicine"[MeSH Terms] OR "telemedicine"[Text Word] OR "mhealth"[Text Word] OR "mobile"[Text Word] OR "ehealth"[Text Word] OR "telehealth"[Text Word] OR "Internet"[MeSH Terms] OR "internet"[Text Word] OR "online"[Text Word] OR "web-based"[Text Word] OR "web based"[Text Word] OR "website*"[Text Word] OR "digital"[Text Word] OR "email*"[Text Word] OR "serious game*" [Text Word] OR "computer*"[Text Word] OR "computer-based"[Text Word] OR "Internet-Based Intervention"[MeSH Terms] OR "Mobile Applications"[MeSH Terms] OR "app"[Text Word] OR "apps"[Text Word] OR "Cell Phone"[MeSH Terms] OR "cell phone*"[Text Word] OR "text-messag*"[Text Word] OR "sms"[Text Word] OR "smart phone*"[Text Word] OR "smartphone*"[Text Word] OR "Social Media"[MeSH Terms] OR "social media"[Text Word] OR "social network*"[Text Word] OR "blog*"[Text Word] OR "forum"[Text Word] OR "YouTube"[Text Word] OR "Snapchat"[Text Word] OR "TikTok"[Text Word] OR "Instagram"[Text Word] OR "Twitter"[Text Word] OR "Discord"[Text Word] OR "Facebook"[Text Word] OR "Reddit"[Text Word] OR "Twitch"[Text Word] OR "Tumblr"[Text Word] OR "WhatsApp"[Text Word] OR "Messenger"[Text Word] OR "WeChat"[Text Word] OR "Telegram"[Text Word] OR "MySpace"[Text Word] OR "podcast*"[Text Word] OR "tablet*"[Text Word] OR "newsletter*"[Text Word])

**AND** (("intervention*"[Text Word] OR "action*"[Text Word] OR "program*"[Text Word] OR "initiative*"[Text Word] OR "project*"[Text Word]) AND ("Health Education"[MeSH Terms] OR "health education"[Text Word] OR "education, health"[Text Word] OR "education program*"[Text Word] OR "Health Promotion"[MeSH Terms] OR "health promotion"[Text Word] OR "promotion, health"[Text Word] OR "promotion program*"[Text Word] OR "Health Communication"[MeSH Terms] OR "health communication*"[Text Word] OR "Primary Prevention"[MeSH Terms] OR "primary prevention*"[Text Word] OR "primordial prevention*"[Text Word] OR "Preventive Health Services"[MeSH Terms] OR "preventive health service*" [Text Word]))

**AND** ("Adolescent"[MeSH Terms] OR "adolescent*"[Text Word] OR "adolescence"[Text Word] OR "youth"[Text Word] OR "youths"[Text Word] OR "teen*"[Text Word] OR "Young Adult"[MeSH Terms] OR "young adult*"[Text Word] OR "young people"[Text Word] OR "young person*"[Text Word] OR "AYA"[Text Word])

**AND** ("evaluat*"[Text Word] OR "outcome*"[Text Word] OR "evaluability"[Text Word] OR "feasibility"[Text Word] OR "acceptability"[Text Word] OR "adherence"[Text Word] OR "engagement"[Text Word] OR "usability"[Text Word] OR "attractiveness"[Text Word] OR "efficacy"[Text Word] OR "effectiveness"[Text Word] OR "impact"[Text Word] OR "side-effect*"[Text Word] OR "side effect*"[Text Word] OR "process evaluation"[Text Word] OR "process result*"[Text Word] OR "implementation"[Text Word] OR "fidelity"[Text Word] OR "mechanism of change"[Text Word] OR "mechanisms of change"[Text Word] OR "economic evaluation"[Text Word] OR "efficiency"[Text Word] OR "cost-effectiveness"[Text Word] OR "cost effectiveness"[Text Word] OR "cost-benefit"[Text Word] OR "cost benefit"[Text Word] OR "cost-utility"[Text Word] OR "cost utility"[Text Word] OR "transferability"[Text Word])

**AND** (("mixed method*"[All Fields] OR "mixed-method*" [All Fields] OR "multimethod*"[All Fields] OR "multi-method*"[All Fields] OR "combined method*"[All Fields] OR "combined approach*"[All Fields] OR "combined process*"[All Fields] OR "composite approach*"[All Fields]) OR (("efficacy"[All Fields] OR "effectiveness"[All Fields] OR "impact"[All Fields] OR "side-effect*"[All Fields] OR "side effect*"[All Fields]) AND ("feasibility"[All Fields] OR "acceptability"[All Fields] OR "adherence"[All Fields] OR "engagement"[All Fields] OR "usability"[All Fields] OR "attractiveness"[All Fields] OR "process evaluation"[All Fields] OR "process result*"[All Fields] OR "implementation"[All Fields] OR "fidelity"[All Fields] OR "mechanism of change"[All Fields] OR "mechanisms of change"[All Fields] OR "economic"[All Fields] OR "efficiency"[All Fields] OR "cost*"[All Fields])) OR ("quantitativ*"[All Fields] AND "qualitativ*"[All Fields])))

**NOT** ("Secondary Prevention"[MeSH Terms] OR "secondary prevention*"[Text Word] OR "Tertiary Prevention"[MeSH Terms] OR "tertiary prevention*"[Text Word])

**NOT** ("RNA, Messenger"[MeSH Terms] OR "messenger RNA"[Text Word])

************************

**CINAHL**

Search history

| Date | Number of references retrieved | Number of duplicates | Number of references added to screen |
| --- | --- | --- | --- |
| February 9^th^ 2023 | 289 | 193 | 96 |
| May 2^nd^ 2023 | 12 | 4 | 8 |
| January 2^nd^ 2024 | 26 | 8 | 18 |
|  | **327** | **205** | **122** |

Search terms

(("telemedicine" OR "mhealth" OR "mobile" OR "ehealth" OR "telehealth" OR "internet" OR "online" OR "web-based" OR "web based" OR "website" OR "digital" OR "email" OR "serious game" OR "computer" OR "computer-based" OR "internet-based intervention" OR "mobile application" OR "app" OR "apps" OR "cell phone" OR "text-messaging" OR "text-message" OR "sms" OR "smart phone" OR "smartphone" OR "social media" OR "social network" OR "blog" OR "forum" OR "YouTube" OR "Snapchat" OR "TikTok" OR "Instagram" OR "Twitter" OR "Discord" OR "Facebook" OR "Reddit" OR "Twitch" OR "Tumblr" OR "WhatsApp" OR "Messenger" OR "WeChat" OR "Telegram" OR "MySpace" OR "podcast" OR "tablet" OR "newsletter")

**AND** (("intervention" OR "action" OR "program" OR "initiative" OR "project") AND ("health education" OR "education program" OR "health promotion" OR "promotion program" OR "health communication" OR "primary prevention" OR "primordial prevention" OR "preventive health services"))

**AND** ("adolescent" OR "adolescence" OR "youth" OR "youths" OR "teen" OR "young adult" OR "young people" OR "young person" OR "AYA")

**AND** ("evaluation" OR "outcome" OR "evaluability" OR "feasibility" OR "acceptability" OR "adherence" OR "engagement" OR "usability" OR "attractiveness" OR "efficacy" OR "effectiveness" OR "impact" OR "side-effect" OR "side effect" OR "process evaluation" OR "process results" OR "implementation" OR "fidelity" OR "mechanism of change" OR "mechanisms of change" OR "economic evaluation" OR "efficiency" OR "cost-effectiveness" OR "cost effectiveness" OR "cost-benefit" OR "cost benefit" OR "cost-utility" OR "cost utility" OR "transferability")

**AND** (("mixed method" OR "mixed-method" OR "multimethod" OR "multi-method" OR "combined method" OR "combined approach" OR "combined process" OR "composite approach") OR (("efficacy" OR "effectiveness" OR "impact" OR "side-effect" OR "side effect") AND ("feasibility" OR "acceptability" OR "adherence" OR "engagement" OR "usability" OR "attractiveness" OR "process evaluation" OR "process result" OR "process results" OR "implementation" OR "fidelity" OR "mechanism of change" OR "mechanisms of change" OR "economic" OR "efficiency" OR "cost")) OR ("quantitative" AND "qualitative")))

**NOT** ("secondary prevention" OR "tertiary prevention")

**NOT** ("messenger RNA")

************************

**EMBASE**

Search history

| Date | Number of references retrieved | Number of duplicates | Number of references added to screen |
| --- | --- | --- | --- |
| February 9^th^ 2023 | 2,344 | 662 | 1,682 |
| May 2^nd^ 2023 | 99 | 16 | 83 |
| January 2^nd^ 2024 | 452 | 226 | 226 |
|  | **2,895** | **904** | **1,991** |

Search terms

('telemedicine'/exp OR 'telemedicine' OR 'mhealth'/exp OR 'mhealth' OR 'mobile' OR 'ehealth'/exp OR 'ehealth' OR 'telehealth'/exp OR 'telehealth' OR 'internet'/exp OR 'internet' OR 'online'/exp OR 'online' OR 'web-based' OR 'web based' OR 'website'/exp OR 'website*' OR 'digital' OR 'email'/exp OR 'email' OR 'serious game'/exp OR 'serious game*' OR 'computer'/exp OR 'computer*' OR 'computer-based' OR 'internet-based intervention'/exp OR 'internet-based intervention*' OR 'mobile application'/exp OR 'mobile application*' OR 'app' OR 'apps' OR 'cell phone'/exp OR 'cell phone*' OR 'text-messaging'/exp OR 'text-messag*' OR 'text-message' OR 'sms' OR 'smart phone'/exp OR 'smart phone*' OR 'smartphone'/exp OR 'smartphone*' OR 'social media'/exp OR 'social media' OR 'social network'/exp OR 'social network*' OR 'blog'/exp OR 'blog*' OR 'forum' OR 'youtube'/exp OR 'youtube' OR 'snapchat'/exp OR 'snapchat' OR 'tiktok'/exp OR 'tiktok' OR 'instagram'/exp OR 'instagram' OR 'twitter'/exp OR 'twitter' OR 'discord' OR 'facebook'/exp OR 'facebook' OR 'reddit'/exp OR 'reddit' OR 'twitch'/exp OR 'twitch' OR 'tumblr'/exp OR 'tumblr' OR 'whatsapp'/exp OR 'whatsapp' OR 'messenger' OR 'wechat'/exp OR 'wechat' OR 'telegram' OR 'myspace'/exp OR 'myspace' OR 'podcast'/exp OR 'podcast*' OR 'tablet'/exp OR 'tablet*' OR 'newsletter'/exp OR 'newsletter*')

**AND** (('intervention'/exp OR 'intervention*' OR 'action'/exp OR 'action*' OR 'program'/exp OR 'program*' OR 'initiative*' OR 'project*') AND ('health education'/exp OR 'health education' OR 'education program'/exp OR 'education program*' OR 'health promotion'/exp OR 'health promotion' OR 'promotion program*' OR 'health communication'/exp OR 'health communication' OR 'primary prevention'/exp OR 'primary prevention' OR 'primordial prevention'/exp OR 'primordial prevention' OR 'preventive health services'/exp OR 'prevention health service*'))

**AND** ('adolescent'/exp OR 'adolescent*' OR 'adolescence'/exp OR 'adolescence' OR 'youth'/exp OR 'youth' OR 'youths' OR 'teen*' OR 'young adult'/exp OR 'young adult*' OR 'young people'/exp OR 'young people' OR 'young person*' OR 'aya')

**AND** ('evaluation'/exp OR 'evaluat*' OR 'outcome'/exp OR 'outcome*' OR 'evaluability' OR 'feasibility'/exp OR 'feasibility' OR 'acceptability'/exp OR 'acceptability' OR 'adherence'/exp OR 'adherence' OR 'engagement'/exp OR 'engagement' OR 'usability'/exp OR 'usability' OR 'attractiveness'/exp OR 'attractiveness' OR 'efficacy'/exp OR 'efficacy' OR 'effectiveness' OR 'impact'/exp OR 'impact' OR 'side-effect'/exp OR 'side-effect*' OR 'side effect'/exp OR 'side effect*' OR 'process evaluation'/exp OR 'process evaluation' OR 'process results'/exp OR 'process result*' OR 'implementation'/exp OR 'implementation' OR 'fidelity'/exp OR 'fidelity' OR 'mechanism of change' OR 'mechanisms of change' OR 'economic evaluation'/exp OR 'economic evaluation' OR 'efficiency'/exp OR 'efficiency' OR 'cost-effectiveness'/exp OR 'cost-effectiveness' OR 'cost effectiveness'/exp OR 'cost effectiveness' OR 'cost-benefit'/exp OR 'cost-benefit' OR 'cost benefit'/exp OR 'cost benefit' OR 'cost-utility'/exp OR 'cost-utility' OR 'cost utility'/exp OR 'cost utility' OR 'transferability'/exp OR 'transferability')

**AND** ('mixed method'/exp OR 'mixed method*' OR 'mixed-method*' OR 'multimethod*' OR 'multi-method*' OR 'combined method*' OR 'combined approach*' OR 'combined process' OR 'composite approach' OR (('efficacy'/exp OR 'efficacy' OR 'effectiveness' OR 'impact'/exp OR 'impact' OR 'side-effect'/exp OR 'side-effect*' OR 'side effect'/exp OR 'side effect*') AND ('feasibility'/exp OR 'feasibility' OR 'acceptability'/exp OR 'acceptability' OR 'adherence'/exp OR 'adherence' OR 'engagement'/exp OR 'engagement' OR 'usability'/exp OR 'usability' OR 'attractiveness'/exp OR 'attractiveness' OR 'process evaluation'/exp OR 'process evaluation' OR 'process results'/exp OR 'process result*' OR 'implementation'/exp OR 'implementation' OR 'fidelity'/exp OR 'fidelity' OR 'mechanism of change' OR 'mechanisms of change' OR 'economic' OR 'efficiency'/exp OR 'efficiency' OR 'cost'/exp OR 'cost')) OR ('quantitative' AND ('qualitative'/exp OR 'qualitative')))

**NOT** ('secondary prevention'/exp OR 'secondary prevention' OR 'tertiary prevention'/exp OR 'tertiary prevention')

**NOT** ('messenger rna'/exp OR 'messenger rna')

************************

**PsycINFO**

Search history

| Date | Number of references retrieved | Number of duplicates | Number of references added to screen |
| --- | --- | --- | --- |
| February 9^th^ 2023 | 1,112 | 109 | 1,003 |
| May 2^nd^ 2023 | 60 | 33 | 27 |
| January 2^nd^ 2024 | 165 | 10 | 155 |
|  | **1,337** | **152** | **1,185** |

Search terms

(e-health or ehealth or digital health or telemedicine or telehealth or internet-based intervention or mhealth or mobile health or m-health or mobile app or mobile application or smartphone application or app or apps or internet or web-based interventions' or 'e-health' or 'internet-based interventions or website or smartphone or online email serious games or video games or digital games or computer or computer-based or computer-based intervention or cellphones or cell phones or sms or text messaging or short message or text messages or smartphone or smart phone or blogs or social media or social network or podcast or podcasts or podcasting or tablet or newsletter

**AND**

initiatives or programs or strategy or intervention or project or action

**AND**

health education or health promotion or promotion or promote or promoting or health communication or communication, health or communications, health or health communications or primary prevention or primordial prevention or preventive health services

**AND**

adolescents or teenagers or young adults or teen or youth or adolescence or young people or young person or youths or teens

**AND**

evaluation methods or evaluation process or evaluation or efficacy or effectiveness or impact or benefits or outcomes or success or efficiency or outcome measures or evaluability or feasibility or acceptability or adherence or engagement or usability or user experience or attractiveness or side effects or adverse effects or process evaluation or implementation evaluation or fidelity of implementation or implementation fidelity or mechanisms of change or economic evaluation or cost economic or economic analysis or cost-effectiveness or cost-benefit or cost-consequence or cost-utility or transferability

**AND**

mixed methods or 'qualitative' and 'quantitative'

**NOT** secondary prevention or secondary disease prevention or tertiary prevention or tertiary education

**NOT** messenger rna)

************************

**ClinicalTrials.gov**

Search history

| Date | Number of references retrieved | Number of duplicates | Number of references added to screen |
| --- | --- | --- | --- |
| January 2^nd^ 2024 | **690** | **31** | **659** |

Search terms

("ehealth" OR "web-based" OR "online" OR "internet" OR "digital" OR "mobile") AND ("promotion" OR "prevention") AND ("adolescent" OR "youth") AND ("evaluation" OR "outcome" OR "feasibility" OR "effectiveness" OR "process" OR "economic")

With filters:

• 10 Years to 25 Years old (🡪 To match eligible interventions)

• Interventional studies (🡪 To identify interventions only)
