# Supplementary material for: Current Practices and a Novel Operational Framework for Planning Research on Digital Health Promotion Interventions From Development to Implementation: Scoping Review
Source: J Med Internet Res. 2026 May 6;28:e82611. doi: 10.2196/82611 (PMC13191305; doi:10.2196/82611)
Supplement: Multimedia Appendix 3 [file jmir_v28i1e82611_app3.docx]

### Multimedia Appendix 3. Data charting form

A more exhaustive data charting form was used for the primary article of this scoping review, notably incorporating data extracted on measured outcomes and their associated results. This form is available in Additional File 3 of the related article:

- Collin C, Eyraud C, Martin P, Michel M, Le Roux E, Alberti C. A scoping review of outcome selection and accuracy of conclusions in complex digital health interventions for young people (2017–2023): methodological proposals for population health intervention research. *BMC Med* 2025;**23**:400. <https://doi.org/10.1186/s12916-025-04245-1>.

| **INTERVENTION-LEVEL CHARACTERISTICS** |
| --- |
| **GENERAL INFORMATION** |
| **Intervention name:** *Text field*  **Registration in a study or trial repository:** □ Yes □ No  **If yes, specify:** □ Clinicaltrials.gov □ ISRCTN □ ANZCTR □ DRKS □ ChiCTR □ Other: *Text field*  **Registration ID:** *Text field*  **Funding source(s):** *Text field*  **Total funding received for intervention development and evaluation:** \|_\|\|_\|\|_\|\|_\|\|_\|\|_\|\|_\|\|_\|  **Currency**: □ EUR □ GBP □ USD □ AUD □ Other: \|_\|\|_\|\|_\|  **Number of published articles related to the intervention**: \|_\|\|_\|  **For each article, specify:** DOI; first author surname; institutional affiliation; year of publication; journal; protocol paper (yes/no) |
| **INTERVENTION DETAILS** |
| **Description:** *Text field*  **Start date (including recruitment):** mm/yyyy  **End date:** mm/yyyy  **Target population:**  □ General AYA (Adolescents and Young Adults) population  □ Specific AYA population. **Specify:** *Text field*  **Age targeted by the intervention.** Lower limit: \|_\|\|_\| years. Upper limit: \|_\|\|_\| years.  **Health topic(s):**  □ Nutrition and physical activity □ Tobacco □ Alcohol  □ Gambling addiction □ Illicit substance uses □ Sexual and reproductive health  □ Vaccination and vaccine-preventable diseases □ Mental health  □ Other: *Text field*  **Planned dose/usage of the intervention:** □ Single exposure □ Repeated exposure □ NR (Not reported)  **If repeated exposure, what is the duration of the exposure?** *Text field*  **If repeated exposure, what is the frequency of the exposure?**  □ As desired by the user □ Other: *Text field* □ NR  **Method(s) used to develop the intervention:**  □ Existing intervention  □ Existing intervention transferred and/adapted to a new population or context  □ New intervention based on theory  □ New intervention based on practice or public policies  □ Other: *Text field* □ NR  **Were participatory methods used in the development of the intervention?** □ Yes □ No  **Does the content of the intervention received vary based on participant characteristics?**  □ No, no personalisation □ Yes, for groups of participants with similar “risks”  □ Yes, individual personalisation (different versions of the intervention for each participant)  □ NR  **Does the intervention content change or evolve during participants’ involvement?**  □ No, content remains fixed throughout participation  □ Yes, content is updated or adapted during participation  □ NR  **Digital technology(ies) used in the intervention:**  **Platform dedicated to the intervention (website, app):** □ Yes □ No □ NR If yes, specify the digital components used: *Text field* (e.g. videos, instant discussions) **Social media (blog, forum, social network, podcast platform):** □ Yes □ No □ NR If yes, specify the social media and components used: *Text field* (e.g. social network name such as Twitter, Snapchat, etc., forums, videos, information messages) **Serious game:** □ Yes □ No □ NR If yes, specify: □ Online/multiplayer game □ Offline/local game **Messaging service (SMS, audio, email, newsletter):** □ Yes □ No □ NR If yes, specify: *Text field* (e.g. SMS, interactive audio) **Telephone call or videoconference service:** □ Yes □ No □ NR If yes, specify: *Text field* (e.g. phone call, Zoom platform)  **Mobile device:** □ Yes □ No □ NR If yes, specify: *Text field* (e.g. tracker, sensor) **Other:** □ Yes □ No If yes, specify: Text field  **Number of intervention components described by the author(s):** \|_\|\|_\| □ NR **Intervention component(s) described by the author(s):** □ Components for disseminating information, education, and promoting specific behaviours (e.g. videos, text messages (SMS, emails, articles, tips), audio messages, graphics, FAQs, etc.) [Passive engagement of the participant] □ Components for standardised interaction between the participant and the digital platform (e.g. interactive voice response, personal goal setting, interactive decision tools, quizzes, etc.) [Active engagement with the platform] □ Components for direct interaction between participants and their peers (live sessions, discussion forums/testimonials, Q&A, phone call, video conference, etc.) [Active engagement with peers through the platform] □ Components for direct interaction between participants and health/promotion professionals (live sessions, discussion forums, Q&A, phone call, video conference, etc.) [Active engagement with professionals through the platform] □ Digital sensor or tracker for data collection/monitoring [No direct engagement of the participant] □ Other: *Text field*  □ NR |

| **PHASE-LEVEL CHARACTERISTICS *(filled in for each research phase)***  □ Development □ Feasibility □ Evaluation (effectiveness) □ Evaluation (process) □ Evaluation(efficiency) □ Implementation □ Other: *Text field* |
| --- |
| **GENERAL INFORMATION** |
| **Number of articles related to the phase: \|_\|**  **DOIs:** ___________________ / ___________________ / ___________________ / ___________________  **Protocol** (if the related article itself has not been published): □ Yes □ No  **Published results:** □ Yes □ No  **Objective of the phase + Label used to describe the phase:** *Text field*  **Start date (including recruitment):** mm/yyyy  **End date:** mm/yyyy  **Is the phase repeated several times (iteration)?** □ Yes □ No  **If yes, describe**: Text field  **Does the phase overlap with another research phase?** □ Yes □ No  **If yes, specify the overlapping phase**: Text field  **If yes, describe**: Text field |
| **STUDY DESIGN** |
| **Which methods are used to conduct in this phase?**  □ Quantitative only □ Qualitative only □ Qualitative + Quantitative □ NR  **If quantitative methods are used:**  **Is this a non-comparative study?** □ Yes □ No  **If yes, what type of study is it?**  □ Case study or case series □ Descriptive cross-sectional study  □ Analytical cross-sectional study □ Cohort study □ Case-control study  □ Time series □ Geographical series □ NR  **Is this a quasi-experimental study?** □ Yes □ No  **If yes, what is the design?**  □ Parallel groups □ Factorial design □ Cross-over □ N-of-1 □ Time-series study  □ Here-Elsewhere (A/B testing) □ Before-and-after (pre-post)  □ MOST (multiphase optimisation strategy)  □ SMART (sequential multiple assignment randomised trial) □ Other: *Text field* □ NR  **Is there any blinding process?** □ Yes □ No □ NR  **Si yes, which?** *(multiple answers)*  □ Participant/Subject □ Researcher □ Statistician □ NR  **Is this an experimental study?** □ Yes □ No  **If yes, what is the design?**  □ Parallel groups □ Factorial design □ Cross-over □ N-of-1  □ Micro-randomised trial (JITAIs)  □ SMART (sequential multiple assignment randomised trial) □ Other: *Text field* □ NR  **Is there any blinding process?** □ Yes □ No □ NR  **Si yes, which?** *(multiple answers)*  □ Participant/Subject □ Researcher □ Statistician □ NR  **At what level is randomisation performed?** □ Individual □ Cluster □ Other: *Text field* □ NR  **Number of allocation arms:** \|_\| □ NR □ NA  **Allocation ratio:** □ Balanced (between arms) □ Unbalanced □ NR  **If qualitative methods are used:**  **On which methods is the evaluation of these domains based?** *(multiple answers)*  □ Focus group(s)      □ Interview(s)      □ Observation(s)      □ Document analysis  □ Questionnaire/commentary free-text field      □ Workshop(s) □ Other: *Text field* □ NR □ NA  **If both qualitative and quantitative methods are used**, **do the authors describe a specific mixed-methods research design?** □ Yes □ No  **If yes, which?** *(multiple answers)*  □ Nested (QUAN (qual) = strengthen the experiment)  □ Explanatory (QUAN → qual = explain results)  □ Convergent (QUAN + QUAL = convergence of results)  □ Exploratory (QUAL → quan = generalise results)  □ Transformative (no formal notation, QUAN → QUAL = highlight inequalities)  □ Multi-phase (QUAN → QUAL → [QUAN + QUAL] = programme development)  □ Other: *Text field* □ NR □ NA  **Other notes on the nature of the study:** *Text field* |
| **AUTHORS’ CONCLUSIONS** |
| **Authors’ conclusions for this research phase:** *Text field*  **Strengths of this research phase as identified by the authors:** *Text field*  **Limitations of this research phase as identified by the authors:** *Text field*  **Did the authors explicitly report the subsequent research phase(s)?** □ Yes □ No  **If yes, how did the authors justify progression to the subsequent research phase(s)?** *Text field*  **Did they use predefined quantitative progression criteria?** □ Yes □ No  **If no, describe the progression mechanism:** *Text field* |
