# Supplementary material for: Current Practices and a Novel Operational Framework for Planning Research on Digital Health Promotion Interventions From Development to Implementation: Scoping Review
Source: J Med Internet Res. 2026 May 6;28:e82611. doi: 10.2196/82611 (PMC13191305; doi:10.2196/82611)
Supplement: Multimedia Appendix 4 [file jmir_v28i1e82611_app4.docx]

### Multimedia Appendix 4. Terminology used to name research phases

**Table 1.** Terminology used by authors to describe digital health interventions’ research program phases mapped against the MRC definition of phases

| **MRC phases** | **Common labels used in identified interventions** | **N*** |
| --- | --- | --- |
| Development or identify | Development/Design | 13 |
| intervention (n = 26) | Formative research process/design and evaluation | 6 |
|  | Co-design/Co-creation/Participatory design study | 5 |
|  | Adaptation | 5 |
|  | Iterative development/design | 4 |
|  | Needs assessment | 4 |
|  | User-design | 1 |
|  | Identification | 1 |
|  | Re-adaptation | 1 |
| Feasibility (n = 23) | Pilot study/Piloting/Pilot project/Pilot testing/Internal | 10 |
|  | Feasibility study | 5 |
|  | Preliminary efficacy | 5 |
|  | Pilot RCT | 2 |
|  | Pre-trial feasibility study | 1 |
|  | Playtesting | 1 |
|  | Beta-testing | 1 |
|  | Formative evaluation | 1 |
| Evaluation (n = 31)^†^ |  |  |
| Effectiveness (n = 31) | Effectiveness | 14 |
|  | Efficacy | 9 |
|  | Impact evaluation | 6 |
|  | Effects | 6 |
|  | Effectiveness-implementation study | 3 |
| Process (n = 26) | Process evaluation | 9 |
|  | Acceptability | 7 |
|  | Engagement | 5 |
|  | Views and experiences | 4 |
|  | Adherence | 3 |
|  | Uptake | 2 |
|  | Exposure | 1 |
|  | Satisfaction | 1 |
|  | Utilization | 1 |
|  | Mediation analysis | 1 |
|  | Moderation analysis | 1 |
| Economic (n = 4) | Cost-effectiveness | 2 |
|  | Budget impact analysis | 2 |
|  | Cost-utility | 1 |
|  | Costs | 1 |
| Implementation (n = 7) | Implementation and recruitment | 2 |
|  | Implementation study | 2 |
|  | Implementation trial | 1 |
|  | Context of implementation | 1 |
|  | Implementation lessons | 1 |

*Counts may not sum to the total number of programs reporting the phase, as some programs used multiple terms to describe a single phase. ^†^The evaluation phase was subcategorized into efficacy or effectiveness, process, and economic evaluations. RCT: randomized controlled trial.
