# Supplementary material for: Current Practices and a Novel Operational Framework for Planning Research on Digital Health Promotion Interventions From Development to Implementation: Scoping Review
Source: J Med Internet Res. 2026 May 6;28:e82611. doi: 10.2196/82611 (PMC13191305; doi:10.2196/82611)
Supplement: Multimedia Appendix 5 [file jmir_v28i1e82611_app5.docx]

**Multimedia Appendix 5. Interrater agreement on classifying phase conduct, phase arrangement, and progression mechanisms for 8 digital health interventions**

**Agreement on phases**

|  |  | **Rater 2 (CE)** | | **Agreement percentage, %** | **Cohen’s kappa, κ** |
| --- | --- | --- | --- | --- | --- |
| **Rater 1 (CC)** | **Development phase** | | | | |
|  |  | Yes | No | 100 | - |
|  | Yes | 8 | 0 |  |  |
|  | No | 0 | 0 |  |  |
|  | **Feasibility phase** | | | | |
|  |  | Yes | No | 100 | - |
|  | Yes | 5 | 0 |  |  |
|  | No | 0 | 3 |  |  |
|  | **Implementation phase** | | | | |
|  |  | Yes | No | 87.5 | 0.71  *(moderate agreement)* |
|  | Yes | 1 | 1 |  |  |
|  | No | 0 | 6 |  |  |

Interrater agreement for the evaluation phase was not calculated, as the presence of an evaluation phase was an eligibility criterion for inclusion in the final sample.

**Agreement on phase arrangements**

*Iterated phases*

|  |  | **Rater 2 (CE)** | | **Agreement percentage, %** | **Cohen’s kappa, κ** |
| --- | --- | --- | --- | --- | --- |
| **Rater 1 (CC)** | **Iterative development** | | | | |
|  |  | Yes | No | 100 | - |
|  | Yes | 3 | 0 |  |  |
|  | No | 0 | 5 |  |  |
|  | **Iterative feasibility** | | | | |
|  |  | Yes | No | 100 | - |
|  | Yes | 0 | 0 |  |  |
|  | No | 0 | 5 |  |  |
|  | **Iterative evaluation** | | | | |
|  |  | Yes | No | 100 |  |
|  | Yes | 1 | 0 |  | - |
|  | No | 1 | 7 |  |  |
|  | **Iterative implementation** | | | | |
|  |  | Yes | No | 87.5 | nr† |
|  | Yes | 0 | 1 |  |  |
|  | No | 0 | 7 |  |  |

†: Cohen’s kappa was not calculated when agreement was very high, but ratings were highly unbalanced across categories, a situation in which κ can be misleading (prevalence paradox). In these cases, percentage agreement is reported instead (1).

*Overlapped phases*

|  |  | **Rater 2 (CE)** | | **Agreement percentage, %** | **Cohen’s kappa, κ** |
| --- | --- | --- | --- | --- | --- |
| **Rater 1 (CC)** | **Overlap: development+feasibility** | | | | |
|  |  | Yes | No | 87.5 | 0.71  *(substantial agreement)* |
|  | Yes | 2 | 0 |  |  |
|  | No | 1 | 5 |  |  |
|  | **Overlap evaluation+implementation** | | | | |
|  |  | Yes | No | 87.5 | nr† |
|  | Yes | 0 | 1 |  |  |
|  | No | 0 | 7 |  |  |
|  | **Overlap: other phases** | | | | |
|  |  | Yes | No | 100 | - |
|  | Yes | 0 | 0 |  |  |
|  | No | 0 | 8 |  |  |

†: Cohen’s kappa was not calculated when agreement was very high, but ratings were highly unbalanced across categories, a situation in which κ can be misleading (prevalence paradox). In these cases, percentage agreement is reported instead (1).

**Agreement on progression mechanisms (n=13)**

|  |  | **Rater 2 (CE)** | | **Agreement percentage, %** | **Cohen’s kappa, κ** |
| --- | --- | --- | --- | --- | --- |
| **Rater 1 (CC)** | **Automatic progression** | | |  |  |
|  |  | Yes | No | 84.6 | 0.68  *(substantial agreement)* |
|  | Yes | 7 | 1 |  |  |
|  | No | 1 | 4 |  |  |
|  | **Conditional progression based on the researchers’ appraisal without prespecified criteria** | | | | |
|  |  | Yes | No | 92.3 | 0.81  *(almost perfect agreement)* |
|  | Yes | 3 | 1 |  |  |
|  | No | 0 | 9 |  |  |
|  | **Conditional progression based on predefined quantitative criteria** | | | | |
|  |  | Yes | No | 100 | - |
|  | Yes | 1 | 0 |  |  |
|  | No | 0 | 12 |  |  |

From: (1) Feinstein AR, Cicchetti DV. High agreement but low Kappa: I. the problems of two paradoxes. *J Clin Epidemiol*. 1990;43(6):543–9.
