# Supplementary material for: Current Practices and a Novel Operational Framework for Planning Research on Digital Health Promotion Interventions From Development to Implementation: Scoping Review
Source: J Med Internet Res. 2026 May 6;28:e82611. doi: 10.2196/82611 (PMC13191305; doi:10.2196/82611)
Supplement: Multimedia Appendix 6 [file jmir_v28i1e82611_app6.docx]

### Multimedia Appendix 6. Hypotheses linking intervention characteristics and research program structure and duration

**Table 1.** Intervention characteristics hypothesized to influence research program structure and/or duration

| **Intervention characteristics** | **Hypothesized influence on program structure** | **Hypothesized influence on program duration** |
| --- | --- | --- |
| **New intervention** (vs adaptation of an existing intervention) | Development phase is less likely to be skipped and more likely to be iterative. | Program likely to be prolonged due to an extended or iterative development phase. |
| **Personalized intervention** (vs "one-size-fits-all"/standardized program) | Early phases are more likely to be iterative. | Program likely to be prolonged due to iterations of early phases. |
| **Intervention with dynamic content** (vs fixed content) | Early phases are more likely to be iterative. | Program likely to be prolonged due to iterations of early phases. |
| **Use of multiple digital technologies** (vs single digital technology) | Early phases are less likely to be skipped and more likely to be iterative, as interoperability of technologies needs to be ensured. | Program likely to be prolonged due to extended early-phase testing. |
| **Use of a behavioral theoretical framework** (e.g. COM-B) **in intervention development** | Development phase is less likely to be iterated, as key behavioral determinants are pre-specified. | Program may be shortened due to a more focused development phase. |
| **Participatory design or development** (vs researcher-led development) | Development phase is more likely to be iterative. Feasibility phase may be skipped, as usability and acceptability are addressed through stakeholder engagement. | Program likely to be prolonged due to iterative development phase.  Program may be shortened as feasibility phase is skipped. |
| **Evaluation within a randomized controlled trial (RCT)** (vs quasi-experimental design) | Feasibility phase is less likely to be skipped, as RCT funding often depends on prior feasibility evidence. | Program likely to be prolonged due to longer evaluation phase timelines (e.g. recruitment, follow-up). |
| **Amount of funding** | Higher funding levels may facilitate the conduct of four-phase research programs and longer evaluation phases. Limited funding may encourage phase overlap. | Programs with greater funding are likely to be prolonged (as four-phase and longer evaluation). Greater funding may reduce delays between phases. |
| **Type of funder** | Public funders are more likely to require adherence to existing guidance, promoting complete four-phase programs. | Publicly funded programs are likely to be prolonged due to complete evaluation requirements. Public-private partnerships may support shorter timelines. |
