# Supplementary material for: Current Practices and a Novel Operational Framework for Planning Research on Digital Health Promotion Interventions From Development to Implementation: Scoping Review
Source: J Med Internet Res. 2026 May 6;28:e82611. doi: 10.2196/82611 (PMC13191305; doi:10.2196/82611)
Supplement: Multimedia Appendix 7 [file jmir_v28i1e82611_app7.docx]

### **Multimedia Appendix 7. Worked examples of digital health research program structures (A–F)**

**Structure A.** Sequential structure only, with automatic progression or progression based on researchers’ appraisal without prespecified criteria (n=6)

***Worked example:*** ARMADILLO intervention


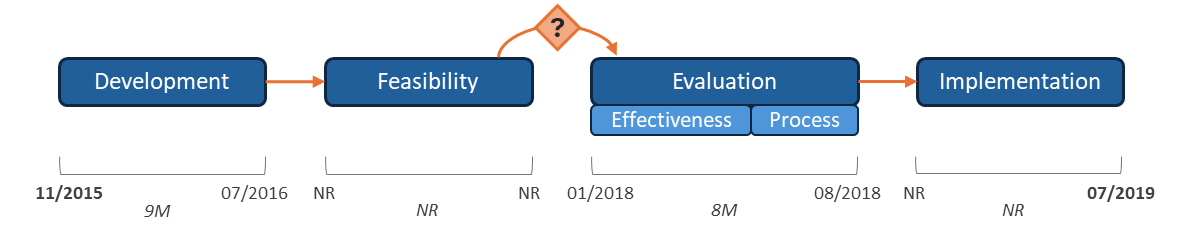


**D** *(auto)* > **F** *(appr)* > **E** *(auto)* > **I**

Gonsalves et al. (2015) described a formative research protocol for the development and piloting of the *ARMADILLO* intervention, which aims to provide sexual and reproductive health information to dispel myths and misconceptions about contraception among young people in Peru and Kenya. The protocol explicitly specified two sequential phases. The first phase comprised formative concept testing to develop intervention content, using focus group discussions (**development phase**). The second phase involved piloting and finalization, consisting of a pre-post feasibility evaluation with baseline assessment of primary and secondary outcomes and usability, a three-week intervention period, and follow-up assessments using the same outcomes, complemented by post-intervention in-depth interviews to assess the feasibility of both the intervention and the evaluation design (**feasibility phase**). These two phases were planned to proceed sequentially and the authors did not report any formal meetings, decision rules, or criteria governing progression from development to feasibility. Progression between these phases was therefore classified as **automatic (*auto*)**. Subsequent publications reporting intervention development (Guerrero et al., 2020) and usability testing (Mwaisaka et al., 2021) were consistent with the original protocol, confirming the classification of both the phase arrangement and the progression mechanism.

In a subsequent protocol, Gonsalves et al. (2018) described an open, three-arm, individually randomized controlled trial evaluating the effectiveness of the developed ARMADILLO intervention (**evaluation phase**). In this paper, the authors detailed how findings from “Stage 1” (development and feasibility) were discussed, interpreted, and appraised by the research team to justify progression to “Stage 2” (the RCT). Because this decision relied on researchers’ qualitative appraisal of prior findings, including usability and positive feedback from users’ experience (Mwaisaka et al, 2021), without prespecified quantitative progression criteria, progression from feasibility to evaluation was classified as **conditional, appraisal-based progression without prespecified criteria (*appr*)**. Evaluation results were later reported by Gichangi et al. (2022) for Kenya and Pérez-Lu et al. (2022) for Peru.

Finally, the 2018 protocol outlined plans for a “Stage 3” study examining real-world uptake following service rollout, corresponding to the **implementation phase**. Outputs from this phase were reported by Gonsalves et al. (2019), focusing on implementation and research lessons learned from the RCTs. Notably, this implementation research was conducted after completion of the evaluation phase and proceeded despite largely negative effectiveness findings from the RCTs. Progression from evaluation to implementation was therefore classified as **automatic (*auto*)**.

Taken together, the ARMADILLO research program exemplifies **Structure A**, defined as a strictly sequential arrangement of development, feasibility, evaluation, and implementation phases, with progression that is either automatic or based on researchers’ appraisal of findings without prespecified criteria.

Associated references:

[19] Gonsalves L, L’Engle KL, Tamrat T, Plourde KF, Mangone ER, Agarwal S, et al. Adolescent/Youth Reproductive Mobile Access and Delivery Initiative for Love and Life Outcomes (ARMADILLO) Study: formative protocol for mHealth platform development and piloting. *Reprod Health* 2015;12:67. <https://doi.org/10.1186/s12978-015-0059-y>.

[20] Guerrero F, Lucar N, Garvich Claux M, Chiappe M, Perez-Lu J, Hindin MJ, et al. Developing an SMS text message intervention on sexual and reproductive health with adolescents and youth in Peru. *Reprod Health* 2020;17:116. <https://doi.org/10.1186/s12978-020-00943-6>.

[21] Gonsalves L, Hindin MJ, Bayer A, Carcamo CP, Gichangi P, Habib N, et al. Protocol of an open, three-arm, individually randomized trial assessing the effect of delivering sexual and reproductive health information to young people (aged 13-24) in Kenya and Peru via mobile phones: adolescent/youth reproductive mobile access and delivery initiative for love and life outcomes (ARMADILLO) study stage 2. *Reprod Health* 2018;15:126. <https://doi.org/10.1186/s12978-018-0568-6>.

[22] Perez-Lu JE, Guerrero F, Cárcamo CP, Alburqueque M, Chiappe M, Hindin MJ, et al. The ARMADILLO text message intervention to improve the sexual and reproductive health knowledge of adolescents in Peru: Results of a randomized controlled trial. *PLOS One* 2022;17:e0262986. <https://doi.org/10.1371/journal.pone.0262986>.

[23] Gichangi P, Gonsalves L, Mwaisaka J, Thiongo M, Habib N, Waithaka M, et al. Busting contraception myths and misconceptions among youth in Kwale County, Kenya: results of a digital health randomised control trial. *BMJ Open* 2022;12:e047426. <https://doi.org/10.1136/bmjopen-2020-047426>.

[24] Gonsalves L, Njeri WW, Schroeder M, Mwaisaka J, Gichangi P. Research and Implementation Lessons Learned From a Youth-Targeted Digital Health Randomized Controlled Trial (the ARMADILLO Study). *JMIR MHealth* *UHealth* 2019;7:e13005. <https://doi.org/10.2196/13005>.

[25] Mwaisaka J, Gonsalves L, Thiongo M, Waithaka M, Sidha H, Alfred O, et al. Young People’s Experiences Using an On-Demand Mobile Health Sexual and Reproductive Health Text Message Intervention in Kenya: Qualitative Study. *JMIR MHealth UHealth* 2021;9:e19109. <https://doi.org/10.2196/19109>.

**Structure B.** Sequential structure only, with progression based on predefined quantitative criteria (n=1)

***Worked example:*** Warna-Warni Waktu intervention


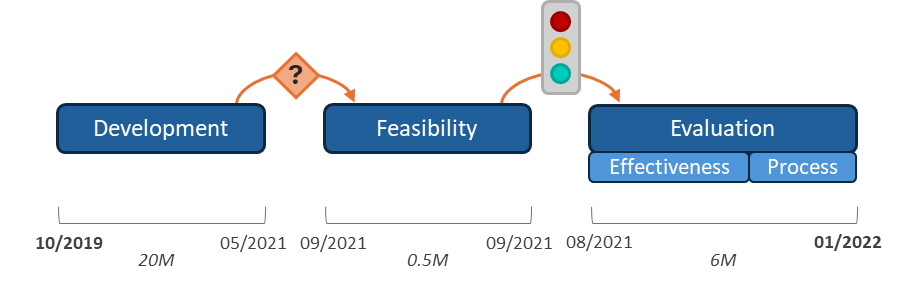


**D** *(appr)* > **F** *(traffic)* > **E**

The development and feasibility phases of the *Warna-Warni Waktu* intervention, which aimed to reduce body dissatisfaction among young Indonesian women, were described by Garbett et al. (2022) in a protocol for a randomized controlled trial (RCT). The **development phase** was conducted between October 2019 and May 2021 and followed a structured, 13-step process combining multiple methods (literature review, online polls, and focus group discussions), engaging more than 300 participants. Acceptability testing was conducted at the end of the development phase, and favorable findings were used by the authors to justify progression to feasibility based on a **qualitative appraisal of findings without prespecified progression criteria (*appr*)**.

The **feasibility phase** was explicitly designed as an internal pilot for the RCT and was analytically and operationally distinct from the subsequent evaluation phase. This phase lasted approximately 0.5 months and employed **prespecified quantitative progression criteria (*traffic*)**, assessed prior to progression to the evaluation phase. Four criteria were defined: (1) participant retention, operationalized as completion of baseline and postintervention assessments, with a ≥70% threshold to proceed (“green”); (2) intervention adherence, defined as the proportion of participants viewing all six intervention videos, with a ≥80% threshold (“green”); (3) data quality, assessed via completion of survey attention checks, with a ≥80% threshold (“green”); and (4) absence of harm, operationalized as no relative worsening in the primary outcome in the intervention arm compared with the control arm between baseline and postintervention assessments.

In a subsequent publication, Garbett et al. (2023) reported that three criteria met the prespecified “green” thresholds, whereas one criterion (intervention adherence) fell within the “amber” category. As prespecified in the protocol, the research team was consulted to determine whether adaptations were required prior to progression. In this case, a modification to intervention delivery was implemented (i.e. the introduction of reminder messages to encourage intervention completion) before proceeding to the full RCT. Progression from feasibility to **evaluation** was therefore classified as conditional progression based on prespecified quantitative criteria, consistent with a traffic-light model. In this structure, predefined quantitative thresholds enable rapid and transparent assessment of progression criteria to inform go/no-go or adapt decisions.

Associated references:

[129] Garbett KM, Craddock N, Haywood S, Nasution K, White P, Saraswati LA, et al. A Novel, Scalable Social Media–Based Intervention (“Warna-Warni Waktu”) to Reduce Body Dissatisfaction Among Young Indonesian Women: Protocol for a Parallel Randomized Controlled Trial. *JMIR Res Protoc* 2022;11:e33596. <https://doi.org/10.2196/33596>.

[130] Garbett KM, Haywood S, Craddock N, Gentili C, Nasution K, Saraswati LA, et al. Evaluating the Efficacy of a Social Media–Based Intervention (Warna-Warni Waktu) to Improve Body Image Among Young Indonesian Women: Parallel Randomized Controlled Trial. *J Med Internet Res* 2023;25:e42499. <https://doi.org/10.2196/42499>.

**Structure C.** Iterative development (n=9), iterative feasibility (n=3), iterative evaluation (n=1) or two-phase iteration (n=1) within a sequential structure, with automatic progression or progression based on researchers’ appraisal without prespecified criteria (n=14)

***Worked example:*** Topity intervention


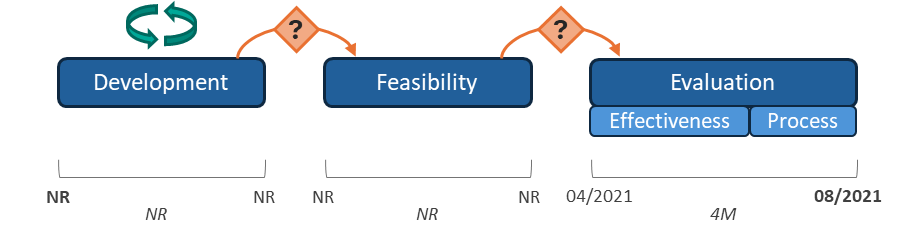


**D(i)** *(appr)* **> F** *(appr)* **> E**

The development and feasibility phases of the *Topity* intervention, aimed at improving body image among Brazilian adolescents, were described by Matheson et al. (2021) in a study protocol for a future randomized trial. The **development phase** followed a four-step process comprising appraisal of existing scientific evidence, collaborative decision-making with partners, selection of therapeutic techniques, and interface development. This process was explicitly described as **iterative (i)**, with repeated refinement informed by collegial discussions among the research team and partners. Progression from development to feasibility was based on these discussions rather than on prespecified quantitative criteria and was therefore classified as **conditional progression based on researchers’ appraisal without prespecified criteria *(appr)***.

Following development, the authors conducted the **feasibility phase** involving 154 adolescents to assess user experience and acceptability, measured across 11 dimensions. Results were reported using descriptive statistics (means and standard deviations), with consistently high ratings across gender and age groups. These findings were used to justify progression to the evaluation phase. However, because the authors did not define explicit acceptability or usability thresholds in advance, progression was again classified as **appraisal-based without prespecified thresholds** ***(appr)***, rather than based on quantitative progression criteria. The authors reported that no substantive adaptations to the intervention were required prior to evaluation. The subsequent **evaluation phase** was conducted as a randomized controlled trial, reported by Matheson et al. (2023).

Associated references:

[127] Matheson EL, Smith HG, Amaral ACS, Meireles JFF, Almeida MC, Mora G, et al. Improving body image at scale among Brazilian adolescents: study protocol for the co-creation and randomised trial evaluation of a chatbot intervention. *BMC Public Health* 2021;21:2135. <https://doi.org/10.1186/s12889-021-12129-1>.

[128] Matheson EL, Smith HG, Amaral ACS, Meireles JFF, Almeida MC, Linardon J, et al. Using Chatbot Technology to Improve Brazilian Adolescents’ Body Image and Mental Health at Scale: Randomized Controlled Trial. *JMIR MHealth UHealth* 2023;11:e39934. <https://doi.org/10.2196/39934>.

**Structure D.** Overlapping development and feasibility phases (with or without iteration) within a sequential structure, with automatic progression or progression based on researchers’ appraisal without prespecified criteria (n=3)

***Worked example:*** Girl2Girl intervention


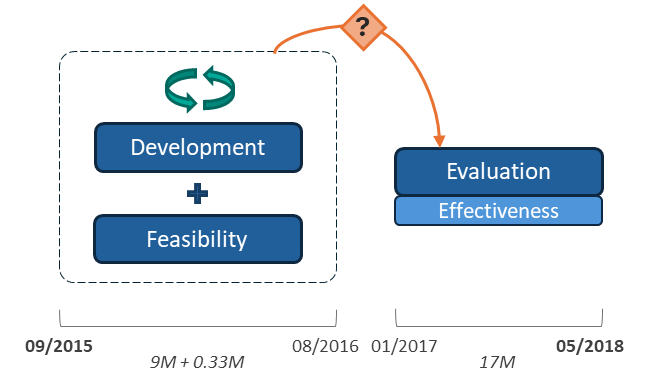


The **development** and **feasibility** phases of the *Girl2Girl* intervention, aimed at preventing adolescent pregnancy among lesbian, gay, bisexual, and other sexual minority (LGB+) cisgender female adolescents, were described by Ybarra et al. (2020). The authors reported four interrelated activities. The first three activities (i.e. focus groups, intervention content writing, and review by a Content Advisory Team (CAT)) were explicitly described as an **iterative** **(i)** process, with repeated refinement of intervention content writing informed by CAT feedback. These activities were classified as part of the development phase.

The fourth activity consisted of beta testing of the developed intervention and was classified as the feasibility phase. Beta testing included piloting the evaluation protocol for a future randomized controlled trial, testing specific program components, and assessing acceptability through a self-safety assessment. Findings from the beta test were subsequently integrated into the intervention, leading to the addition of new components, reprogramming of existing elements, and development of additional messages. As a result, development and feasibility activities were conducted in parallel and informed each other, and were therefore classified as **overlapping** development and feasibility phases with iteration within development ([D(i)+F]).

Based on positive feasibility findings and the refinements made during beta testing, but without prespecified quantitative progression criteria, the authors justified progression to a full-scale randomized controlled trial using a qualitative appraisal of findings. Progression from feasibility to evaluation was therefore classified as **conditional progression based on researchers’ appraisal without prespecified criteria *(appr)***. The **evaluation phase** results are subsequently presented in Ybarra et al. (2020), with a one-year follow-up presented in Ybarra et al. (2023).

Associated references:

[52] Ybarra ML, Price-Feeney M, Prescott T, Goodenow C, Saewyc E, Rosario M. Girl2Girl: How to develop a salient pregnancy prevention program for cisgender sexual minority adolescent girls. *J Adolesc* 2020;85:41–58. <https://doi.org/10.1016/j.adolescence.2020.09.006>.

[53] Ybarra M, Goodenow C, Rosario M, Saewyc E, Prescott T. An mHealth Intervention for Pregnancy Prevention for LGB Teens: An RCT. *Pediatrics* 2021;147:e2020013607. <https://doi.org/10.1542/peds.2020-013607>.

[54] Ybarra M, Rosario M, Saewyc E, Goodenow C, Dunsiger S. One-Year Follow-up After a Pregnancy Prevention Intervention for LGB1 Teens: An RCT. *Pediatrics* 2023;151:e2022059172. <https://doi.org/10.1542/peds.2022-059172>.

[55] Ybarra ML, Saewyc E, Rosario M, Dunsiger S. Subgroup Analyses of Girl2Girl, a Text Messaging-Based Teen Pregnancy Prevention Program for Sexual Minority Girls: Results from a National RCT. *Prev Sci* 2023;24:292–9. <https://doi.org/10.1007/s11121-023-01493-6>.

**Structure E.** Overlapping evaluation and implementation phases (with or without iteration) within a sequential structure, with automatic progression or progression based on researchers’ appraisal without prespecified criteria (n=6)

***Worked example:*** CyberRwanda intervention


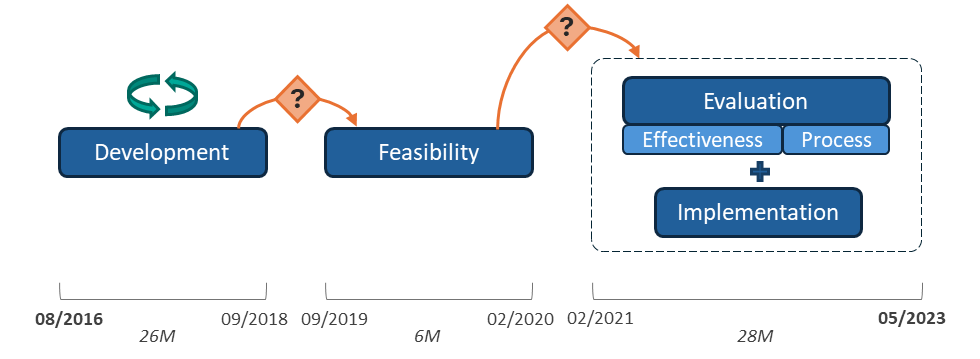


**D(i)** *(appr)* > **F** *(appr)* > **[E+I]**

The overall research program of the *CyberRwanda* intervention, aimed at improving family planning and reproductive health among young people, was described by Ippoliti et al. (2021) using a phased timeline: Phase I, Discovery (August 2016–August 2017); Phase II, Test and Iterate (September 2017–September 2018); Phase III, Pilot and Implementation (September 2018–June 2023); and Phase IV, Launch at Scale (July 2023–September 2024). In alignment with the MRC definition of the development phase, Phases I and II were grouped into a single, extended **development phase**, intended to produce an initial version of the intervention. As described by the authors, this phase was **iterative (i)**, involving repeated cycles of development, usability testing, redevelopment, and retesting.

Following positive development findings, **appraised by the research team without prespecified progression criteria *(appr)***, a pilot study was conducted between September 2019 and February 2020, as reported by Hémono et al. (2022), followed by approximately 11 months of post-pilot adaptation. The pilot, categorised as the **feasibility phase**, assessed acceptability, feasibility, and engagement, primarily using qualitative in-depth interviews alongside quantitative engagement measures. No prespecified quantitative progression thresholds were reported. Based on **researchers’ appraisal of these findings (*appr*)**, the authors concluded that the intervention was acceptable and feasible for both participants and stakeholders, thereby justifying progression to the next phase.

The subsequent phases were described by Nolan et al. (2020) as a protocol for a hybrid type 2 effectiveness–implementation trial, designed to simultaneously evaluate intervention effectiveness and compare two implementation strategies. The explicit use of this hybrid design led to classification of the **evaluation** and **implementation** phases as **overlapping** **([E+I])**. This hybrid trial was conducted over approximately 28 months between 2021 and 2023, consistent with the originally planned timeline, with results reported in Hémono et al. (2024a, 2024b, 2025).

Associated references:

[35] Nolan C, Packel L, Hope R, Levine J, Baringer L, Gatare E, et al. Design and impact evaluation of a digital reproductive health program in Rwanda using a cluster randomized design: study protocol. *BMC Public Health* 2020;20:1701. <https://doi.org/10.1186/s12889-020-09746-7>.

[36] Ippoliti N, Sekamana M, Baringer L, Hope R. Using Human-Centered Design to Develop, Launch, and Evaluate a National Digital Health Platform to Improve Reproductive Health for Rwandan Youth. *Glob Health Sci Pract* 2021;9:S244–60. <https://doi.org/10.9745/GHSP-D-21-00220>.

[37] Hémono R, Packel L, Gatare E, Baringer L, Ippoliti N, McCoy SI, et al. Digital self-care for improved access to family planning and reproductive health services among adolescents in Rwanda: preliminary findings from a pilot study of CyberRwanda. *Sex Reprod Health Matters* 2022;29:2110671. <https://doi.org/10.1080/26410397.2022.2110671>.

[38] Hémono R, Gatare E, Kayitesi L, Packel L, Hunter LA, Kunesh J, et al. CyberRwanda’s Pathway to Impact: Results From a Cluster-Randomized Trial of Adolescent Family Planning Knowledge, Beliefs, Self-Efficacy, and Behavior. *J Adolesc Health* 2024;74:1239–48. <https://doi.org/10.1016/j.jadohealth.2024.01.035>.

[39] Hémono R, Gatare E, Kayitesi L, Hunter LA, Packel L, Ippoliti N, et al. Effect of a digital school-based intervention on adolescent family planning and reproductive health in Rwanda: a cluster-randomized trial. *Nat Med* 2024;30:3121–28. <https://doi.org/10.1038/s41591-024-03205-1>.

[40] Hémono R, Hunter LA, Gatare E, Kayitesi L, Bagwaneza T, Umutoni R, et al. Exposure and Engagement Drive Impact: Results From a Large-Scale Trial of a Digital Family Planning and Reproductive Health Intervention in Rwanda. *J Adolesc Health* 2025;77:947–57. <https://doi.org/10.1016/j.jadohealth.2025.06.039>.

**Structure F.** Multiple overlaps within a sequential structure, with progression based on researchers’ appraisal without prespecified criteria (n=1)

***Worked example:*** Aim2Be intervention


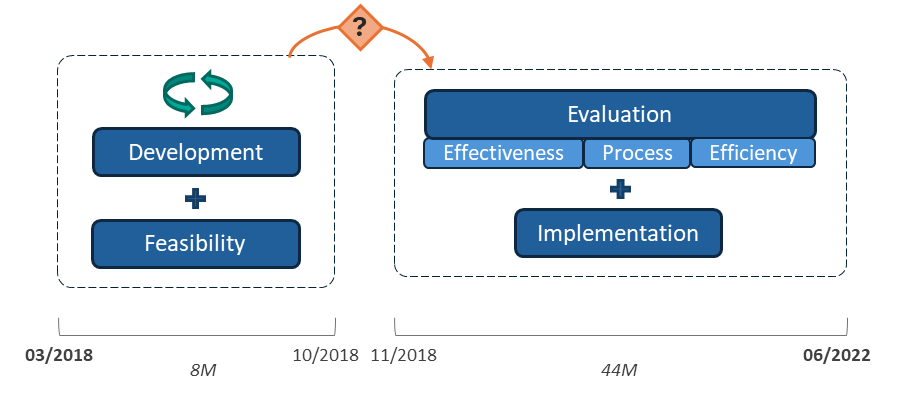


**[D(i)+F]** *(appr)* > **[E+I]**

The development and feasibility phases of the Aim2Be intervention, aimed at promoting health behaviors (including physical activity, nutrition, screen time, and sleep) among adolescents with overweight or obesity, were described by Mâsse et al. (2020) in a protocol for a future randomized controlled trial. The **development phase** was described as an **iterative (i)** process that resulted in a refined version of the intervention, which was then piloted over 4.5 months with 301 adolescents and 301 parents. This pilot was classified as the **feasibility phase** and consisted of a quantitative assessment of preliminary efficacy alongside semi-structured interviews to evaluate usability.

Findings from the feasibility study informed additional development activities, leading to further refinement of the intervention. Because the authors reported a shared overall timeline of approximately eight months for development and feasibility activities, and described iterative feedback loops between these activities, the development and feasibility phases were classified as **overlapping** (**[D(i)+F]**). Based on positive findings from both phases, particularly with respect to preliminary efficacy, progression to the next phase was justified. However, in the absence of prespecified quantitative progression criteria, progression was classified as **conditional progression based on researchers’ appraisal without prespecified criteria (*appr*)**.

A subsequent randomized controlled trial was conducted between November 2018 and June 2022 to evaluate intervention effectiveness and was classified as the **evaluation phase**, as reported in Tugault-Lafleur et al. (2023). During this trial, the authors also collected data on implementation strategies, including assessment of alternative recruitment approaches to enhance reach and uptake. Findings from these **implementation**-related components were reported by Buckler et al. (2023). Because evaluation and implementation activities were conducted concurrently, these phases were classified as **overlapping** (**[E+I]**).

Associated references:

[8] Piatkowski C, Faulkner GE, Guhn M, Mâsse LC. User Characteristics and Parenting Practices Associated with Adolescents’ Initial Use of a Lifestyle Behavior Modification Intervention. *Child Obes* 2020;16:367–78. <https://doi.org/10.1089/chi.2020.0035>.

[9] Lin Y, Mâsse LC. A look at engagement profiles and behavior change: A profile analysis examining engagement with the Aim2Be lifestyle behavior modification app for teens and their families. *Prev Med Rep* 2021;24:101565. <https://doi.org/10.1016/j.pmedr.2021.101565>.

[10] Mâsse LC, Vlaar J, Macdonald J, Bradbury J, Warshawski T, Buckler EJ, et al. Aim2Be mHealth intervention for children with overweight and obesity: study protocol for a randomized controlled trial. *Trials* 2020;21:132. <https://doi.org/10.1186/s13063-020-4080-2>.

[11] Tugault-Lafleur CN, De-Jongh González O, Macdonald J, Bradbury J, Warshawski T, Ball GDC, et al. Efficacy of the Aim2Be Intervention in Changing Lifestyle Behaviors Among Adolescents With Overweight and Obesity: Randomized Controlled Trial. *J Med Internet Res* 2023;25:e38545. <https://doi.org/10.2196/38545>.

[12] De-Jongh González O, Tugault-Lafleur CN, Buckler EJ, Hamilton J, Ho J, Buchholz A, et al. The Aim2Be mHealth Intervention for Children With Overweight or Obesity and Their Parents: Person-Centered Analyses to Uncover Digital Phenotypes. *J Med Internet Res* 2022;24:e35285. <https://doi.org/10.2196/35285>.

[13] Deslippe AL, González OD-J, Buckler EJ, Ball GDC, Ho J, Bucholz A, et al. Do Individual Characteristics and Social Support Increase Children’s Use of an MHealth Intervention? Findings from the Evaluation of a Behavior Change MHealth App, Aim2Be. *Child Obes* Print 2022. <https://doi.org/10.1089/chi.2022.0055>.

[14] Buckler EJ, González OD-J, Ball GDC, Hamilton J, Ho J, Morrison KM, et al. Recruiting families using social media versus pediatric obesity clinics: A secondary analysis of the Aim2Be RCT. *Contemp Clin Trials* 2023;133:107322. <https://doi.org/10.1016/j.cct.2023.107322>.
