# Supplementary material for: Current Practices and a Novel Operational Framework for Planning Research on Digital Health Promotion Interventions From Development to Implementation: Scoping Review
Source: J Med Internet Res. 2026 May 6;28:e82611. doi: 10.2196/82611 (PMC13191305; doi:10.2196/82611)
Supplement: Multimedia Appendix 9 [file jmir_v28i1e82611_app9.docx]

### Multimedia Appendix 9. Description of intervention characteristics and hypothesis testing

**Table 1.** Description of digital health intervention characteristics (n=31)

| **Intervention characteristics** | **N** |
| --- | --- |
| Number of studied interventions | 31 |
| New intervention | 19 |
| Adapted intervention | 12 |
| Personalized intervention | 21 |
| Standardized intervention | 10 |
| Dynamic content | 5 |
| Fixed content | 26 |
| Multiple technology | 13 |
| Single technology | 18 |
| Theory-driven intervention | 21 |
| Empirically-driven intervention | 7 |
| Not reported | 3 |
| Participatory development | 11 |
| Not participatory development | 15 |
| Not applicable | 5 |
| Evaluation study design |  |
| Randomized controlled trial (RCT) | 28 |
| Quasi-experimental design | 3 |
| Funding, in USD |  |
| <500k | 4 |
| 500k-1M | 4 |
| 1-5M | 5 |
| >5M | 3 |
| Not reported | 15 |
| Funder |  |
| Public | 20 |
| Private | 5 |
| Mixed (public and private) | 3 |
| Not reported | 3 |

USD: United States dollar; k: thousands; M: million.

**Table 2.** Description of intervention characteristics in relation to program structure (n=31)

| **Intervention characteristics** | **N** | **Research program structure**  n (row%) | | | **Iterative development**  n (row%) | **Iterative early phases**  n (row%) |
| --- | --- | --- | --- | --- | --- | --- |
|  |  | 2-phase | 3-phase | 4-phase |  |  |
| Number of studied interventions | 31 | 10 | 16 | 5 | 17 | 20 |
| New intervention | 19 |  |  |  | 12 (63) | 15 (79) |
| Adapted intervention | 12 |  |  |  | 5 (42) | 5 (42) |
| Personalized intervention | 21 |  |  |  |  | 13 (62) |
| Standardized intervention | 10 |  |  |  |  | 7 (70) |
| Dynamic content | 5 |  |  |  |  | 4 (80) |
| Fixed content | 26 |  |  |  |  | 16 (62) |
| Multiple technology | 13 |  |  |  |  | 9 (69) |
| Single technology | 18 |  |  |  |  | 11 (61) |
| Theory-driven intervention | 21 |  |  |  | 13 (62) |  |
| Empirically-driven intervention | 7 |  |  |  | 4 (57) |  |
| Not reported | 3 |  |  |  | 0 (0) |  |
| Participatory development | 11 |  |  |  | 7 (64) |  |
| Not participatory development | 15 |  |  |  | 10 (67) |  |
| Not applicable | 5 |  |  |  | 0 (0) |  |
| Evaluation study design |  |  |  |  |  |  |
| Randomized controlled trial (RCT) | 28 |  |  |  |  |  |
| Quasi-experimental design | 3 |  |  |  |  |  |
| Funding, in USD |  |  |  |  |  |  |
| <500k | 4 | 2 (50) | 2 (50) | 0 (0) |  |  |
| 500k-1M | 4 | 2 (50) | 2 (50) | 0 (0) |  |  |
| 1-5M | 5 | 1 (20) | 4 (80) | 0 (0) |  |  |
| >5M | 3 | 0 (0) | 6 (33) | 2 (67) |  |  |
| Not reported | 15 | 5 (33) | 7 (47) | 3 (20) |  |  |
| Funder |  |  |  |  |  |  |
| Public | 20 | 7 (35) | 11 (55) | 2 (10) |  |  |
| Private | 5 | 1 (33) | 1 (33) | 1 (33) |  |  |
| Mixed (public and private) | 3 | 1 (20) | 2 (40) | 2 (40) |  |  |
| Not reported | 3 | 1 (33) | 2 (67) | 0 (0) |  |  |

Grey cells indicate the absence of a priori hypotheses linking intervention characteristics with program structure. See Multimedia Appendix 6 for the full set of hypotheses.

USD: United States dollar; k: thousands; M: million.

**Table 3.** Description of intervention characteristics in relation to program duration (n=31)

| **Intervention characteristics** | **N** | **Total program duration**  n* | | | **Evaluation phase duration**  n (row%) | | |
| --- | --- | --- | --- | --- | --- | --- | --- |
|  |  | < 4 years | 4-7 years | > 7 years | < 12 months | 12-24 months | > 24 months |
| Number of studied interventions | 31 | 4 | 7 | 2 | 13 | 9 | 8 |
| New intervention | 19 | 3 | 6 | 1 |  |  |  |
| Adapted intervention | 12 | 1 | 1 | 1 |  |  |  |
| Personalized intervention | 21 | 2 | 4 | 2 |  |  |  |
| Standardized intervention | 10 | 2 | 3 | 0 |  |  |  |
| Dynamic content | 5 | 0 | 1 | 0 |  |  |  |
| Fixed content | 26 | 4 | 6 | 2 |  |  |  |
| Multiple technology | 13 | 0 | 5 | 1 |  |  |  |
| Single technology | 18 | 4 | 2 | 1 |  |  |  |
| Theory-driven intervention | 21 | 4 | 5 | 0 |  |  |  |
| Empirically-driven intervention | 7 | 0 | 2 | 1 |  |  |  |
| Not reported | 3 | 0 | 0 | 1 |  |  |  |
| Participatory development | 11 | 2 | 3 | 1 |  |  |  |
| Not participatory development | 15 | 2 | 3 | 0 |  |  |  |
| Not applicable | 5 | 0 | 1 | 1 |  |  |  |
| Evaluation study design |  |  |  |  |  |  |  |
| Randomized controlled trial (RCT) | 28 | 4 | 6 | 2 | 11 (39) | 9 (32) | 7 (25) |
| Quasi-experimental design | 3 | 0 | 1 | 0 | 2 (67) | 0 (0) | 1 (33) |
| Funding, in USD |  |  |  |  |  |  |  |
| <500k | 4 | 1 | 1 | 0 | 3 (75) | 1 (25) | 0 (0) |
| 500k-1M | 4 | 0 | 1 | 0 | 2 (50) | 1 (25) | 1 (25) |
| 1-5M | 5 | 0 | 1 | 1 | 0 (0) | 2 (40) | 3 (60) |
| >5M | 3 | 1 | 0 | 0 | 0 (0) | 1 (33) | 2 (67) |
| Not reported | 15 | 2 | 4 | 1 | 8 (53) | 4 (27) | 2 (13) |
| Funder |  |  |  |  |  |  |  |
| Public | 20 | 3 | 3 | 1 |  |  |  |
| Private | 5 | 0 | 2 | 0 |  |  |  |
| Mixed (public and private) | 3 | 1 | 1 | 0 |  |  |  |
| Not reported | 3 | 0 | 1 | 1 |  |  |  |

*Row percentages were not calculated for total program duration because of small sample sizes within each category.

Grey cells indicate the absence of a priori hypotheses linking intervention characteristics with program duration. See Multimedia Appendix 6 for the full set of hypotheses.

USD: United States dollar; k: thousands; M: million.
